# Spatiotemporal observations of host-pathogen interactions in mucosa during SARS-CoV-2 infection indicate a protective role of ILC2s

**DOI:** 10.1128/spectrum.00878-23

**Published:** 2023-11-08

**Authors:** Wei Hu, Lu Meng, Chao Wang, Wenhan Lu, Xiaoyu Tong, Rui Lin, Tao Xu, Liang Chen, An Cui, Xiaoqing Xu, Anni Li, Jia Tang, Hongru Gao, Zhenle Pei, Ruonan Zhang, Yicong Wang, Yu Wang, Wendong Han, Ning Jiang, Chenglong Xiong, Yi Feng, Kuinyu Lee, Mingquan Chen

**Affiliations:** 1 Department of Emergency Medicine, Shanghai Key Laboratory of Infectious Diseases and Biosafety Emergency Response, National Medical Center for Infectious Diseases, Huashan Hospital, Fudan University, Shanghai, China; 2 Department of Infectious Diseases, Shanghai Key Laboratory of Infectious Diseases and Biosafety Emergency Response, National Medical Center for Infectious Diseases, Huashan Hospital, Fudan University, Shanghai, China; 3 Department of Integrative Medicine and Neurobiology, School of Basic Medical Sciences, Institutes of Brain Science, Brain Science Collaborative Innovation Center, State Key Laboratory of Medical Neurobiology, Institute of Acupuncture and Moxibustion, Fudan Institutes of Integrative Medicine, Fudan University, Shanghai, China; 4 The Center for Microbes, Development and Health, Key Laboratory of Molecular Virology and Immunology, Institute Pasteur of Shanghai, Chinese Academy of Sciences, Shanghai, China; 5 State Key Laboratory of Genetic Engineering, School of Life Science, Fudan University, Shanghai, China; 6 Department of Infectious Diseases, National Medical Center for Infectious Diseases, Shanghai Key Laboratory of Infectious Diseases and Biosafety Emergency Response, Huashan Hospital, Shanghai Medical College, Fudan University, Shanghai, China; 7 State Key Laboratory of Cardiovascular Disease, Fuwai Hospital, National Center for Cardiovascular Diseases, Chinese Academy of Medical Sciences and Peking Union Medical College, Beijing, China; 8 Biosafety Level 3 Laboratory, Shanghai Medical College Fudan University, Shanghai, China; 9 Department of Epidemiology, School of Public Health, and Key Laboratory of Public Health Safety, Ministry of Education, School of Public Health, Fudan University, Shanghai, China; Indian Institute of Science, Bangalore, Karnataka, India

**Keywords:** SARS-CoV-2, ILC2, mucosal immunity, tissue transparency and 3D imaging

## Abstract

Innate lymphoid cells (ILCs) play a crucial role in mucosal protection and tissue homeostasis. However, the early mucosal defense mechanisms against the severe acute respiratory syndrome coronavirus 2 (SARS-CoV-2) virus have not been fully understood to date. This knowledge gap has motivated us to develop a coronavirus disease 2019-like mouse model to investigate the interactions between the virus, its receptor, and ILCs. In our study, we conducted intranasal challenges using pseudovirus expressing Spike (PSV-S) to examine the expression patterns of humanized ACE2 (chiACE2) and the transduction of PSV-S in lung and ileum tissues over a specified time period. Within a span of 72 hours, we observed an intense viral transduction induced by the spike protein, which was detected from the central bronchus to the bronchiole and alveolus in the lung (levels 3 to 8). Similarly, we observed the transduction of PSV-S in the villi and crypts of the ileum. Interestingly, our precise three-dimensional colocalization analysis revealed that only 73.74% ± 1.76% of the PSV-S transduction depended on chiACE2. Furthermore, type 2 innate lymphoid cells (ILC2s) exhibited the most pronounced activation in the gut mucosa. This finding indicates the significant role of ILC2s as contributors to the mucosal defense against viral infections. Our data shed light on the critical involvement of ILC2s and their intricate three-dimensional regulatory network in combating SARS-CoV-2 within the mucosa.

**IMPORTANCE:**

Our study revealed the spatial interaction between humanized ACE2 and pseudovirus expressing Spike, emphasizing the role of type 2 innate lymphoid cells during the initial phase of viral infection. These findings provide a foundation for the development of mucosal vaccines and other treatment approaches for both pre- and post-infection management of coronavirus disease 2019.

## INTRODUCTION

The outbreak of the novel severe acute respiratory syndrome coronavirus 2 (SARS-CoV-2) in December 2019 and its Delta and Omicron variants have infected nearly 620 million people worldwide and have had devastating global health, social, and economic effects (1, 2). Therefore, there is an urgent need for more research on the mechanisms, treatments, and prevention as well as vaccination development for the highly contagious coronavirus disease 2019 (COVID-19) disease (3). Although fever occurs in most cases, COVID-19’s clinical manifestations vary from moderate to severe respiratory failure (4) and involve multiple organ systems, and therefore, it is considered to be a systemic disease (5 – 7). The most common initial manifestations include pneumonia (19% ~ 50% of cases), gastroenteritis (59.7% of cases), stomatitis (68% of cases), Smell and taste abnormalities (52% of cases and 44% of cases), conjunctivitis (1.1% of cases), dermatitis (20.4% of cases), and lower urinary tract symptoms (49.8% of cases), indicating that the target of viral attacks is the external interface of the host and that mucosal lesions are a common feature regardless of the site of infection (8 – 14). In addition, lung and intestinal mucosal lesions are the two most frequent and earliest host sites of invasion, and in severe cases, these occur almost simultaneously (7). However, the exact physical barriers and immune defenses against SARS-CoV-2 infection remain largely unknown.

The mucosal system is present from the lung through the intestine as a continuation of embryonic development, and it includes more peripheral tissues such as the epithelium of the oropharynx, urogenital tract, and conjunctiva. Importantly, the mucosal immune response forms a primary barrier against pathogens (15, 16). In general, there are two types of immunity, with type 1 primarily defending against intracellular pathogens such as viruses and type 2 playing a protective role during allergic inflammation and parasitic infection (17, 18). In the case of SARS-CoV-2, the association between viral spike protein and host angiotensin-converting enzyme II (ACE2) functions as the main entrance point into cells, and this leads to an innate immune response in the local mucosa (19, 20). Toll-like and nucleotide-binding oligomerization-like receptors function as two important innate sensors of viral particles and subsequently trigger the expression of type 1 cytokines such as interleukins (IL-1β, IL-2, IL-6, IL-7, and IL-10), interferon γ (IFN-γ), and tumor necrosis factor α (TNF-α) (21, 22). However, type 2 cytokines such as IL-4, IL-13, and IL-33 have also recently been found to be associated with severe COVID-19 along with highly abundant type 2 innate lymphoid cells (ILC2s) in the mucosa (23 – 28). ILC2s are a highly diverse group of cells that play crucial roles in regulating tissue homeostasis and repair as well as in the regulation of other type 2 immune cells (29). Recent clinical findings for this particular ILC subset (30) make it evident that the normal roles of type 1 and type 2 immune cells have been challenged by this novel pathogen (31).

To explore the mucosal immune responses, we first generated a humanized ACE2 (chiACE2) mouse model (32 – 34) and infected them with a pseudovirus expressing Spike (PSV-S) carrying the SARS-CoV-2 spike proteins, which are among the most determinate virulence factors. The spatiotemporal patterns of chiACE2 and PSV-S in both the lung and gut mucosal systems were examined together with the mucosa-specific ILCs. Briefly, the three-dimensional (3D) patterns appeared similar and were characterized by the induction of chiACE2 and the accumulation of PSV-S in the mucosa during the early phase of the infection. Furthermore, ILC classification and cytokine analysis revealed the important protective effects of ILC2 and unconventional type 2 immunity. Thus, our results provide a panoramic view in terms of virus-receptor interactions and mucosal immune defenses and provide a foundation for the development of inhaled vaccines and other potential treatments by enhancing mucosal immunity.

## RESULTS

### Spatiotemporal interactions between spike and chiACE2

**Fig 1:**
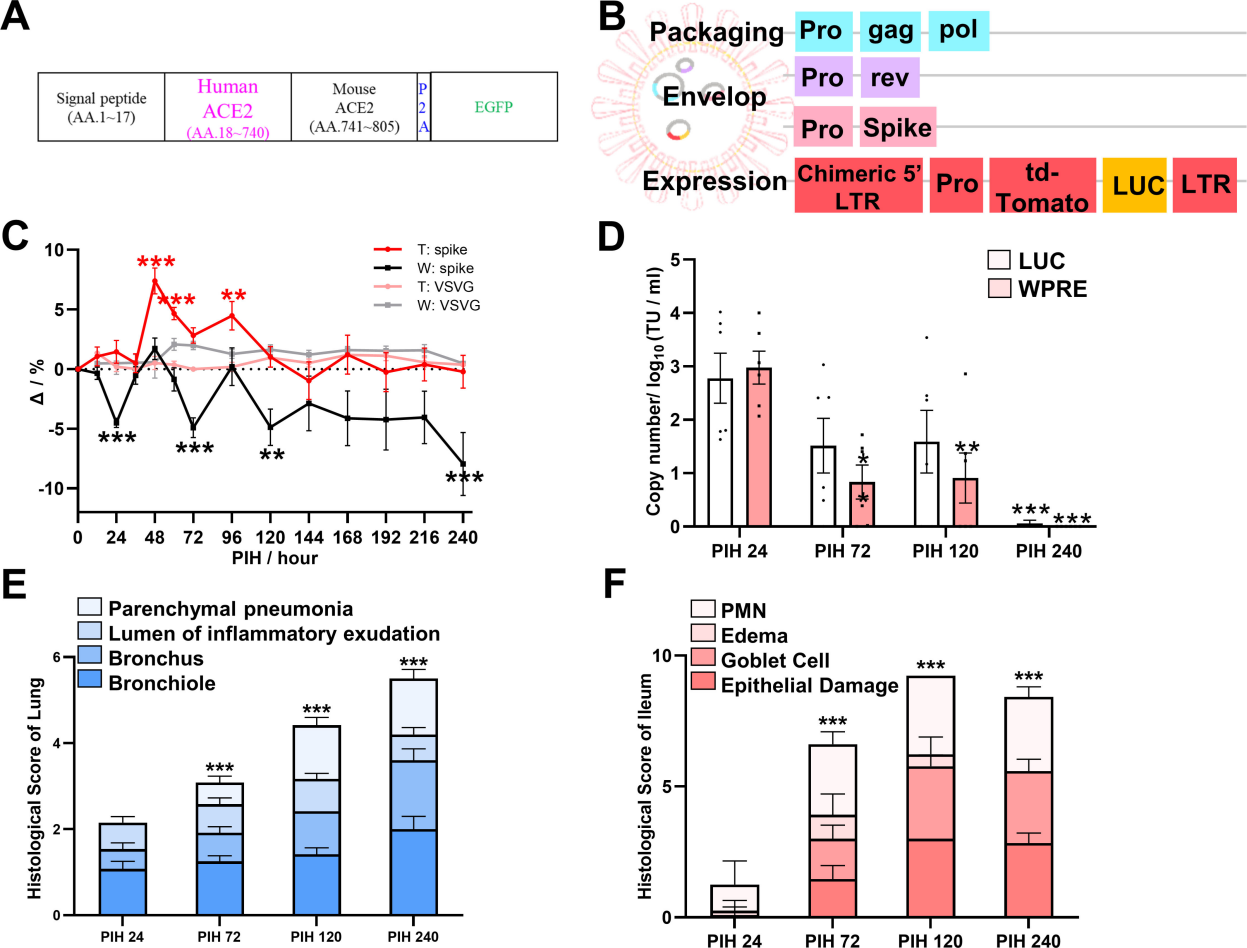
The COVID-19-like chiACE2 mouse model exhibited lung and gut lesions. (**A**) Schematic diagram of the chiACE2 transgenic strategy showing the humanized coding DNA sequence of mouse ACE2. (**B**) Schematic diagram for creating the PSV-S showing spike protein expressed in the envelop components encoded by the VSVG plasmid. (**C**) Temperature and body weight of chiACE2 mice change over time following infection. The baseline measurements of temperature and body weight were recorded prior to infection. To establish a control group for PSV-S infection, we utilized chiACE2 transgenic mice treated with VSVG. T, temperature; W, weight. (**D**) Copy number of PSV-S. TU/mL, transduction units per milliliter; LUC, Luciferase; WPRE, Woodchuck Hepatitis Virus Posttranscriptional Regulatory Element. (**E, F**) Histological scores for HE staining of the lung and ileum. Data are shown as means ± SEM, *n* = 6/group, *P < 0.05, **P < 0.01, and ***P < 0.001.

We first established a humanized ACE2 mouse model using the CRISPR/Cas9 knockin technology. To achieve this (Fig. 1A; detailed information about amino acid and gene sequence can be seen in Table S1), we replaced the mouse ACE2 extracellular region with the human ACE2 extracellular region (AA.18 ~ 740), which is located in the GRC m38.p6 sites on chromosome X. This genetic modification disrupted the mouse *mAce2* gene and effectively terminated its expression. In addition, we inserted the EGFP gene downstream of the chimera ACE2 (chiACE2) using a P2A linker (GSGATNFSLLKQAGDVEENPGP), enabling the co-expression of chiACE2 and EGFP. To introduce the targeting construct, including the subgenomic RNA (sgRNA) and Cas9 mRNA, we performed microinjections into zygotes of C57BL/6 mice. The successful insertion of the construct was confirmed by sequencing analysis. Subsequently, the F0 mice were backcrossed with C57BL/6 mice, and the heterozygous offspring were further verified through PCR screening using specific primers (forward: 5′-CGACAACCACTACCTGAGCAC-3′, reverse: 5′-GTGAAGATGTCCACTCAAGTCTGA-3′).

Utilizing the co-expression strategy, we investigated the overall expression patterns of chiACE2 by examining EGFP expression in lung sections of the chiACE2 mice. Consequently, we successfully developed a stable humanized ACE2 mouse model, termed chiACE2 mice, in which chiACE2 (comprising the extracellular domain of human ACE2 and the intracellular domain of mouse ACE2) was expressed under the regulation of the *mACE2* promoter. This targeted approach facilitated an inherent expression profile of chiACE2 under the control of the original *Ace2* promoter.

A pseudovirus was engineered to carry the SARS-CoV-2 spike protein using an HIV-based lentivirus system. The construction involved the transfection of three specific plasmids: pMD2.G-SARS-COV-2 Spike envelope, psPAX2 packaging plasmid, and GPLVX-CMV-tdTomato-T2A-LUC (See Materials and Methods for details). Following intranasal infection with pseudovirus expressing Spike (PSV-S) (Fig. 1B), we observed a COVID-19-like syndrome characterized by various symptoms such as increased body temperature, decreased body weight, and reduced cecal weight (Fig. 1C; Fig. S3A) (35). To confirm viremia, we examined the serum for the presence of the PSV-S, which was detected (Fig. 1D). Additionally, during specimen collection, we observed several gross morphological and pathological changes, including splenomegaly, renal hemorrhage, and swollen lymph nodes (Fig. S3B). Furthermore, severe damage to the lungs and intestines was evident, while other organs such as the liver, spleen, and kidney exhibited varying degrees of damage. Specifically, the lung tissue displayed parenchymal pneumonia and shedding of the bronchial stratified ciliated epithelium as the main changes, while the ileum showed goblet cell proliferation and neutrophil infiltration, thus confirming the validity of our COVID-19-like mouse model (Fig. 1E and F; Fig. S4).

As the binding of the spike protein to ACE2 is recognized as the primary cellular entry point, we aimed to further investigate the specific 3D interactions between spike and chiACE2. Given that the lung and intestines were the organs most profoundly affected and impacted at early stages, we focused on monitoring these interactions in those particular organs (Video S1 and S2).

Notably, we observed no binding of the PSV-S in wild-type (WT) C57BL/6 mice administered with vesicular stomatitis virus G (VSVG) nor in WT or chiACE2 transgenic mice treated with phosphate-buffered saline (PBS) alone. Similarly, no binding was observed in VSVG-chiACE2 transgenic mice (Fig. S5). These findings provide evidence that the specific binding observed between spike and ACE2 was exclusive to the experimental conditions involving chiACE2 expression and PSV-S administration.

**Fig 2:**
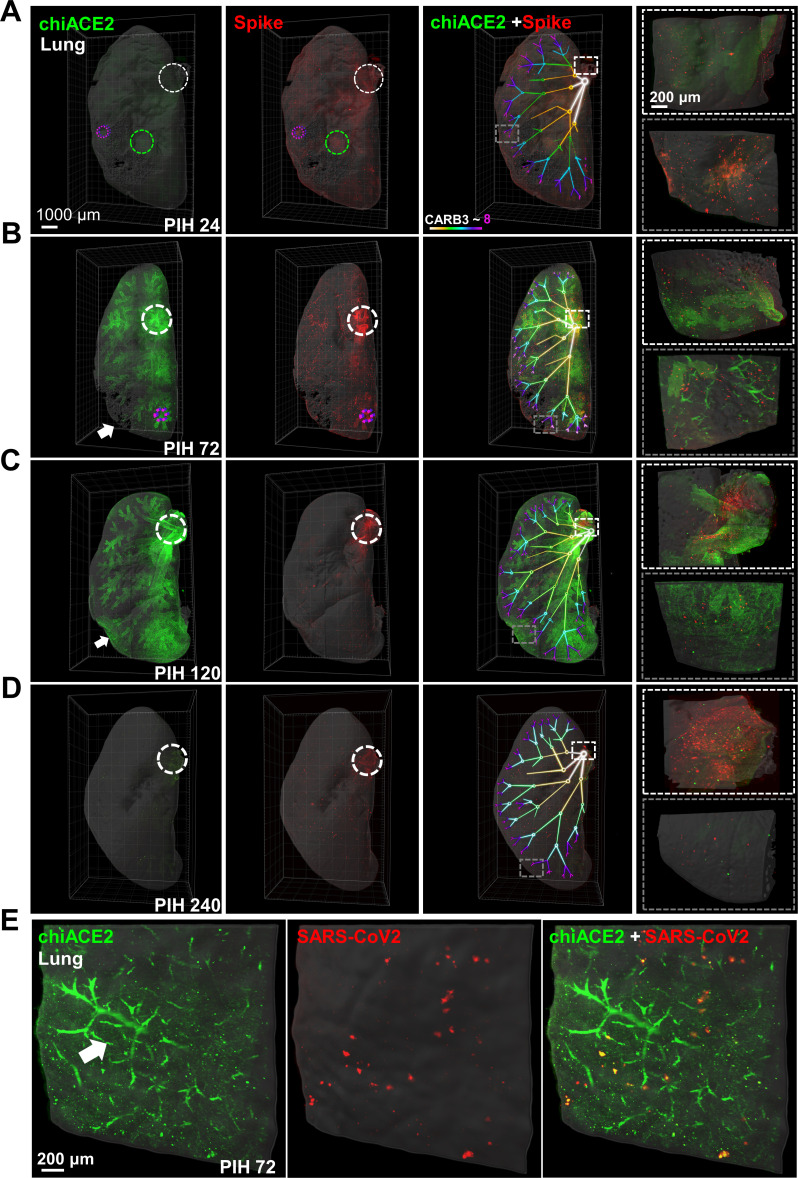
Spatiotemporal changes in chiACE2-spike interactions in the lungs of the COVID-19-like mouse model. (**A–D**) Representative chiACE2-spike spatiotemporal interactions in the lung during PIH 24–240. The circle in the first two columns indicates the PSV-S foci, with the color corresponding to the level of the bronchus and the arrow indicating the peripheral infiltration and vasculature. The dendrograms in the third column show the different levels of the bronchus, and the fourth column shows the magnified views from the square regions in the third column. (**E**) Induced chiACE2 bound to SARS-CoV-2 accumulated in the bronchus and periphery alveolus. The clearly outlined peripheral microvasculature clusters in the SARS-Cov-2-infected model appeared at PIH 72, similar to PSV-S-simulated infection.

In the lung (Fig. 2A through D), we observed invasion and induced expression of chiACE2 from post-infection hour (PIH) 24 to 120. Given the highly stratified nature of the bronchi, we conducted further analysis by reconstructing and categorizing different levels of the bronchi based on their maximum/minimum diameters (Table S4). This resulted in the identification of six levels, ranging from CaRMB3 (Caudal Lobe Right Main Bronchus 3) to CaRMB8. We found that chiACE2 expression was rapidly induced from the central bronchus (CaRMB3-4) to the peripheral alveoli and this induction was synchronized with the spike infection process. However, it is worth noting that only a few chiACE2 foci were found to be co-localized with the PSV-S. Intriguingly, we observed an induction of chiACE2 expression in the aggregated microvascular epithelium at PIH 72. Furthermore, SARS-CoV2 particles exhibited more consistent co-localization with chiACE2 in the bronchus and peripheral alveolus (Fig. 2E). Remarkably, the SARS-CoV2 spike protein clearly demonstrated co-localization with chiACE2 in both the bronchus and peripheral alveolus.

**Fig 3:**
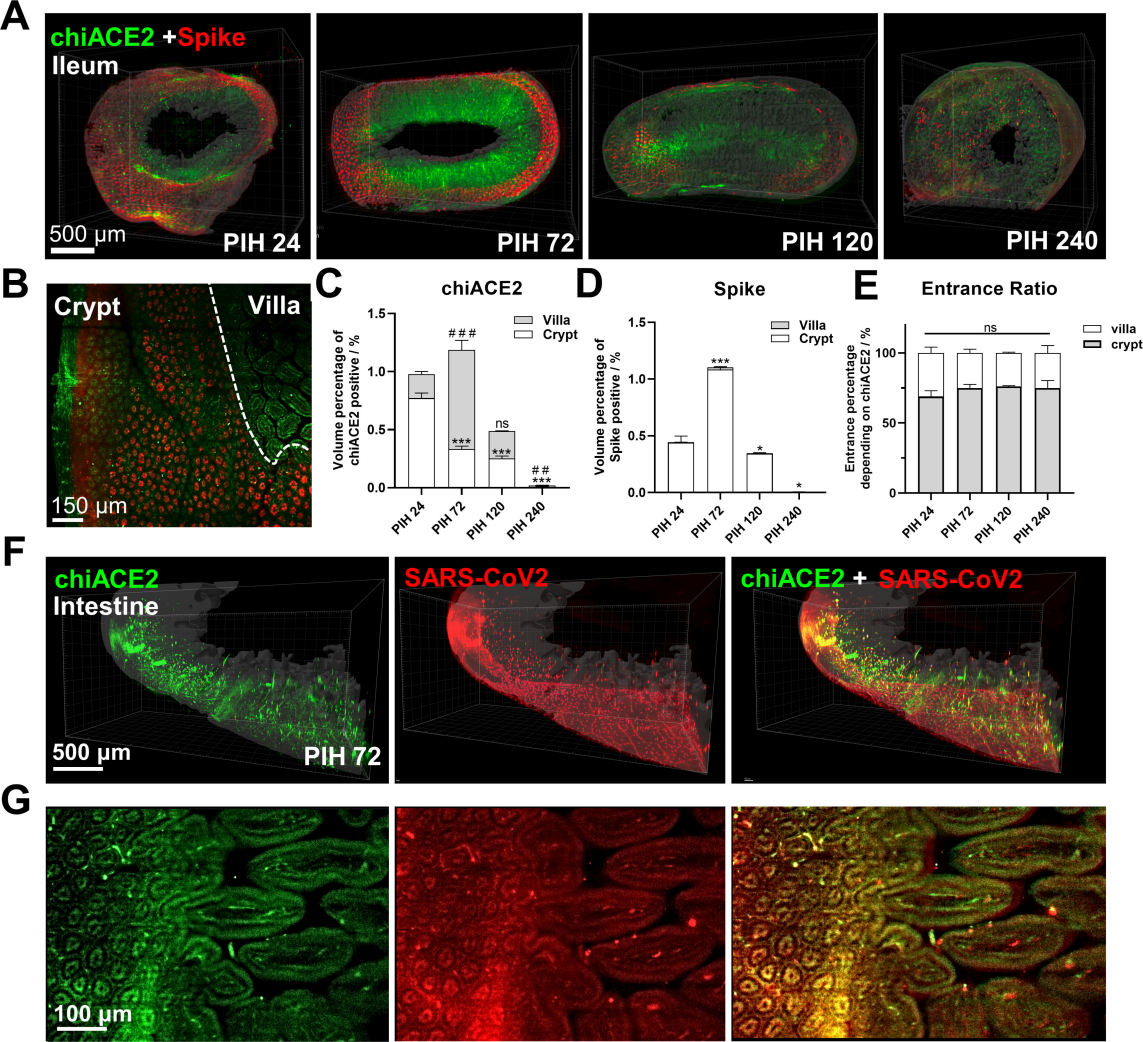
Spatiotemporal changes in chiACE2-spike interactions in the ileum of the COVID-19-like mouse model. (**A**) Representative chiACE2-spike interactions in the ileum during PIH 24–240. (**B**) A perpendicular slice through the villa showing spike bound to chiACE2 in the crypt but not in the villa. (**C, D**) Quantification of chiACE2 and spike in the villa and crypt. (**E**) Entrance ratio depends on chiACE2 of PSV-S in crypts or in villa, calculated as the total chiACE2 divided by spike-bound ACE2. (**F**) Induced chiACE2 bound to SARS-CoV-2 accumulated in the villa and crypt. (**G**) The clearly outlined villa and crypt appeared similar after SARS-CoV-2 infection. Data are shown as means ± SEM, *n* = 3/group, *P < 0.05, **P < 0.01, and ***P < 0.001.

Similar spatiotemporal patterns for spike and chiACE2 were also observed in the ileum. Spike invasion occurred from PIH24 to PIH72, accompanied by chiACE2 induction (Fig. 3A; Fig. S6). However, the duration of spike invasion and chiACE2 induction in the ileum was shorter compared with that in the lung. The binding of spike protein clearly delineated the crypt and villous epithelium, which represented the two primary localizations of spike and chiACE2 (Fig. 3B). Precise 3D quantitative analysis revealed that chiACE2 expression was predominantly observed in the villi (Fig. 3C), while spike mainly accumulated in the crypts (Fig. 3D). Notably, our investigation of the interaction between spike and chiACE2 revealed that spike invasion relied on ACE2 in approximately 73.74% ± 1.76% of crypts and almost 100% of villi, underscoring the crucial role of ACE2 as the primary entry point (Fig. 3E). Additionally, in the SARS-CoV-2 infection model, we observed extensive expression of chiACE2 in the villi epithelium in the ileum, along with the presence of dispersed unbound SARS-CoV-2 particles, which appeared to be less prominent in the PSV-S model (Fig. 3F and G).

### The protective role of ILC2s in the pulmonary and intestinal mucosa

**Fig 4:**
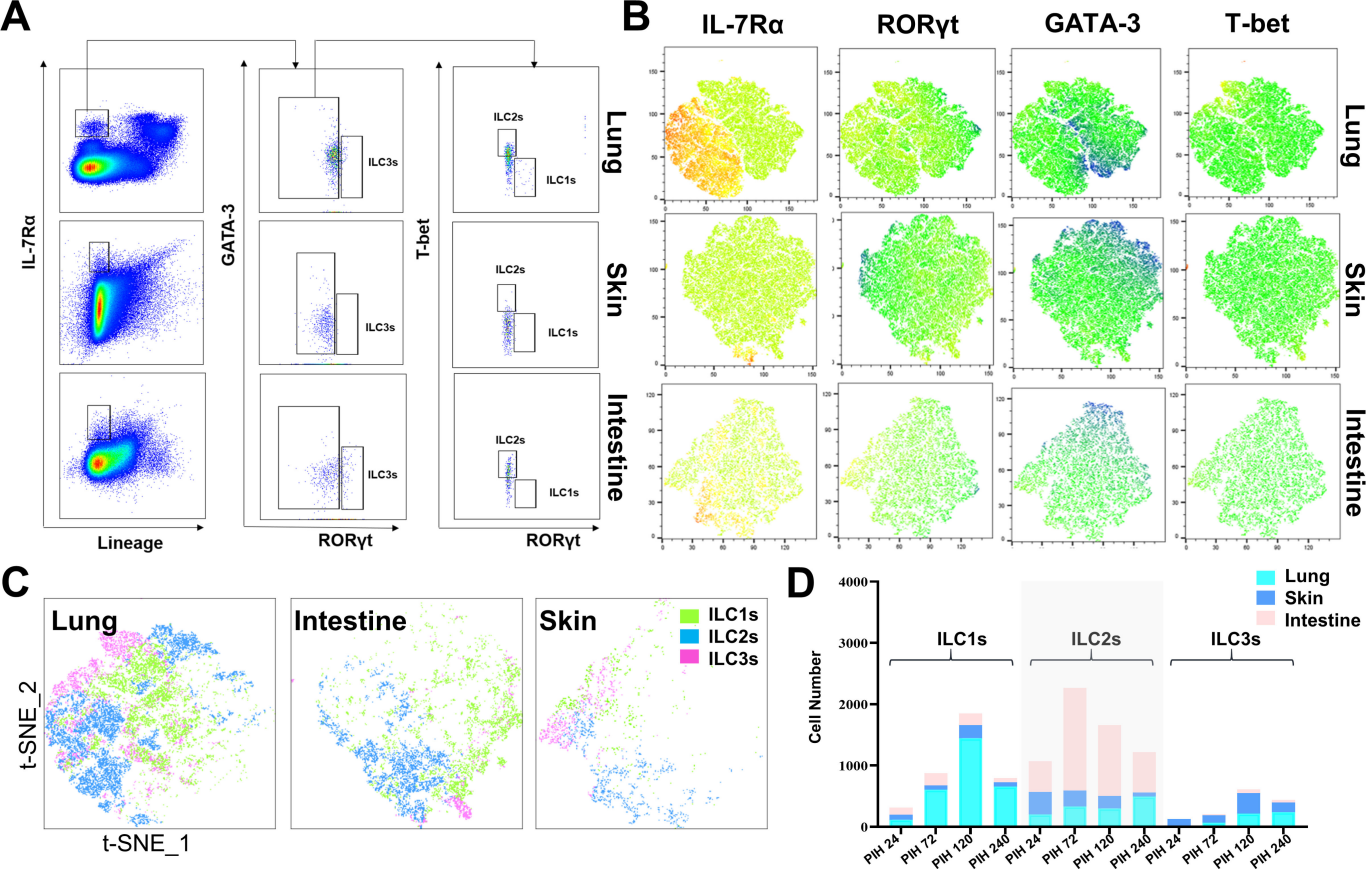
ILC distribution in the pulmonary and intestinal mucosa of the COVID-19-like mouse model. (**A**) Gating strategy for multicolor flow cytometry to identify three types of ILCs. (**B**) t-SNE showing the classification markers for ILCs, including IL-7Rα, RORγt, GATA-3, and T-bet. (**C**) t-SNE showing the total ILCs together with ILC1s, ILC2s, and ILC3s separately. (**D**) Changes in the amounts of the three ILC groups over time post-infection. Data are shown as means ± SEM, *n* = 6/group, *P < 0.05, **P < 0.01, and ***P < 0.001.

Due to the crucial role of innate lymphoid cells (ILCs) in bridging the gap between innate and adaptive immune responses, as well as their reported correlation with SARS-CoV-2 infection (28, 36), our study aimed to investigate the presence of these ILCs at the vulnerable mucosal interface in the lungs and intestines. In our study, mouse innate lymphoid cells were characterized as IL-7Rα^+^/Lin^–^ cells, and the expression of RORγt, GATA-3, and T-bet was analyzed to identify the three ILC groups (Fig. 4A and B). We separately characterized ILC populations in different nonlymphoid tissues, including the lung, intestine, and skin, at various post-infection time points: PIH 24, 72, 120, and 240. Utilizing t-SNE (t-distributed stochastic neighbor embedding) representation, we observed substantial differences in the proportions of ILC subtypes following PSV-S infection in these three tissues (Fig. 4C). The trends in ILC populations were consistent among the lung, intestine, and skin (Fig. 4D; Fig. S7).

Furthermore, we noted a significant increase in ILC2s during the early phase (PIH 72) in both the lung and intestine (Fig. 4D; Fig. S7). In detail, ILC1 levels exhibited higher expression in the lungs, followed by ILC2s and ILC3s. Conversely, in the intestine, ILC2 levels were found to be more prominent, followed by ILC1s and ILC3s. In contrast, ILC3s exhibited increased levels during the late phase (PIH 120–240), while it persisted as the smallest subset throughout this period. These findings suggested that distinct sub-populations of ILCs were recruited to different tissues during PSV-S infection.

### The increase in ILC2s was found to be associated with the concurrent increase in goblet and tuft cells

Due to the significant enrichment of ILC2s in both the lung and intestine, we further explored their associated cells. Previous research has demonstrated that tuft cell-derived IL-25 regulates intestinal ILC2 and goblet cells in the epithelial response circuit during parasitic infections (37). To investigate this further, we performed AB-PAS (Alcian Blue PAS) staining, which revealed that ILC2s were closely associated with acidic mucin-secreting goblet cells in both the lung and intestinal mucosa (Fig. S8). Additionally, we examined the associated tuft cells and observed an increase in their presence at PIH 24–72 in both the lung and gut, particularly in the trachea and ileal epithelium (Fig. S9).

**Fig 5:**
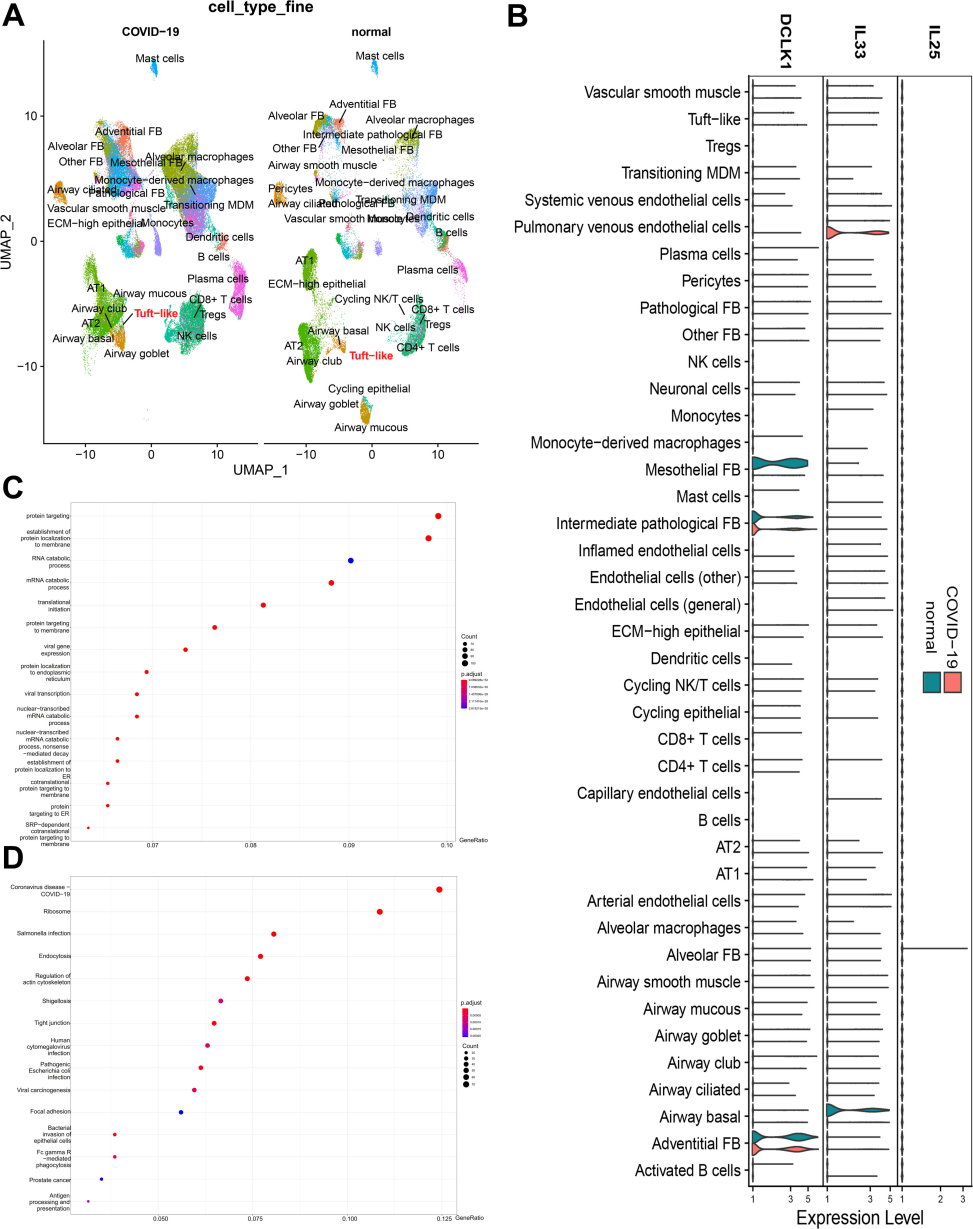
Reanalysis of scRNA-seq data in lung tissue from COVID-19 patients. (**A**) Overview of the major cell types from COVID-19 patients compared with controls in UMAP (data source: https://singlecell.broadinstitute.org/single_cell/study/SCP1219). (**B**) Violin plots of *DCLK1*, *IL-25*, and *IL-33* expression in the assigned cell clusters. (**C, D**) GO and KEGG analysis revealed important relationships between tuft cells and viral infection.

Moreover, a re-analysis of scRNA-seq data obtained from the lungs of COVID-19 patients (38) showed an elevation in tuft-like and goblet cells in these individuals (Fig. 5A). This finding suggests that ILC2s, along with their potentially interacting cells such as goblet cells and tuft cells, collectively play a defensive role against SARS-CoV-2 invasion during the early phase of infection in the lung and intestine.

Taking into consideration that tuft cells play a role in type 2 immunity and cholinergic regulation (39, 40), we conducted further investigations into neuroimmune signaling. Regarding the interaction between tuft cells and ILC2s, we tested two common signaling molecules and found that IL-25 underwent time-dependent expression in the villi, while IL-33 seemed to have a little relationship with tuft cells, as shown in Fig. S10. Moreover, the re-analyzed data from COVID-19 patients indicated that there was no noticeable bias in the expression of IL-25, IL-33, or other activation molecules related to ILC2s in tuft cells (Fig. 5B through D; Fig. S11). Furthermore, we delved into the realm of neuroimmune crosstalk by examining changes in sympathetic nerves (tyrosine hydroxylase+), cholinergic nerves (choline acetyltransferase, ChAt+), and neurons (Tuj1+ or NeuN+). Interestingly, we made the intriguing observation that cholinergic nerves approached tuft cells, implying the presence of mutual interactions between them (Fig. S12).

**Fig 6:**
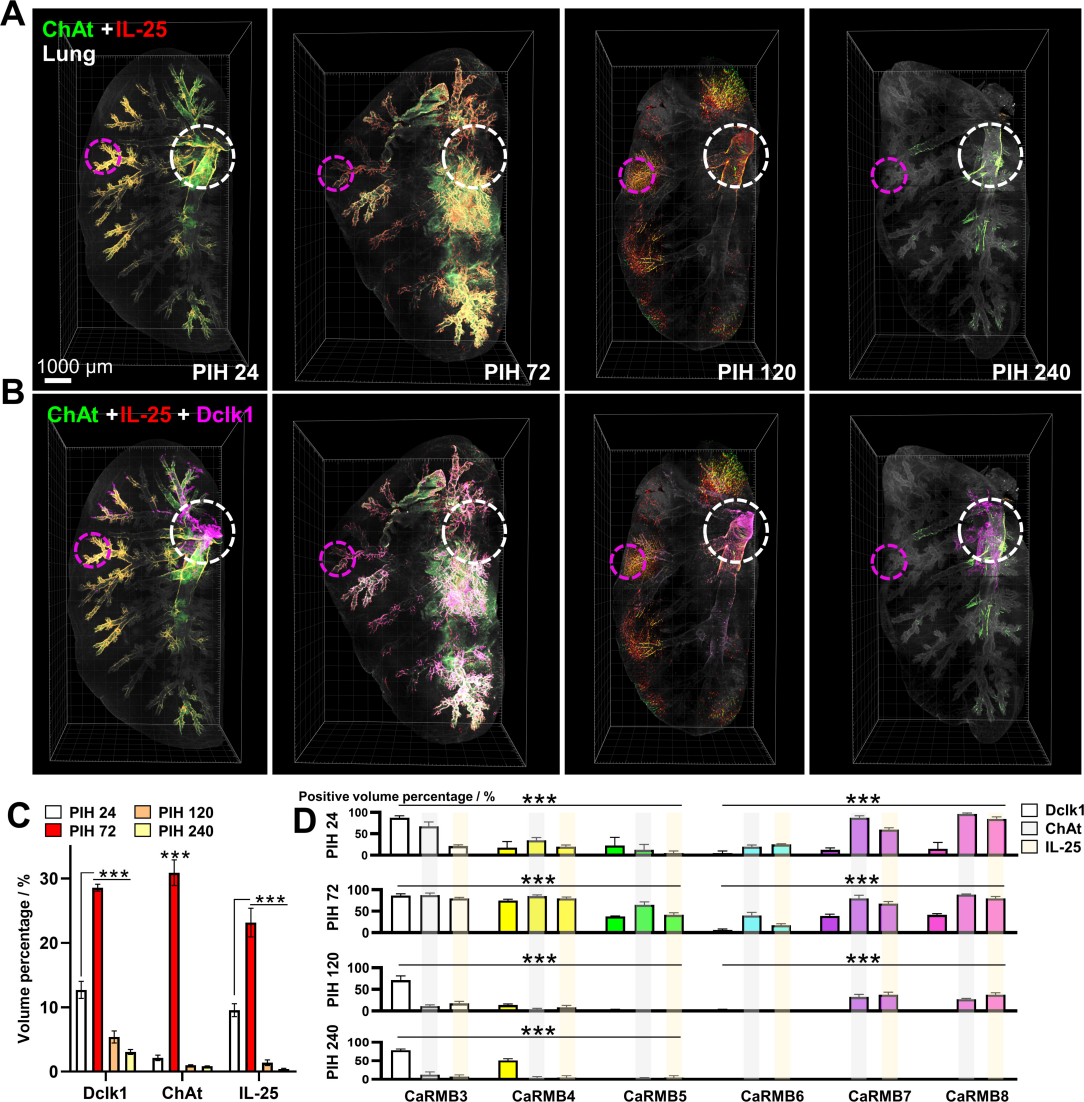
ILC2s correlated with tuft cells and cholinergic innervations in the lungs of the COVID-19-like mouse model. (**A, B**) Representative spatiotemporal changes in cholinergic nerves, tuft cells, and IL-25 in the lung from PIH 24 to PIH 240, with the white circles indicating the central bronchus area and the pink circles indicating the ending of the bronchiole based on the bronchus dendrogram level. (**C**) 3D quantification of Dclk1, ChAt, and IL-25. (**D**) Quantitative analysis of Dclk1, ChAt, and IL-25 changes according to bronchial level. Data are shown as means ± SEM, *n* = 3/group, **P < 0.01 and ***P < 0.05.

**Fig 7:**
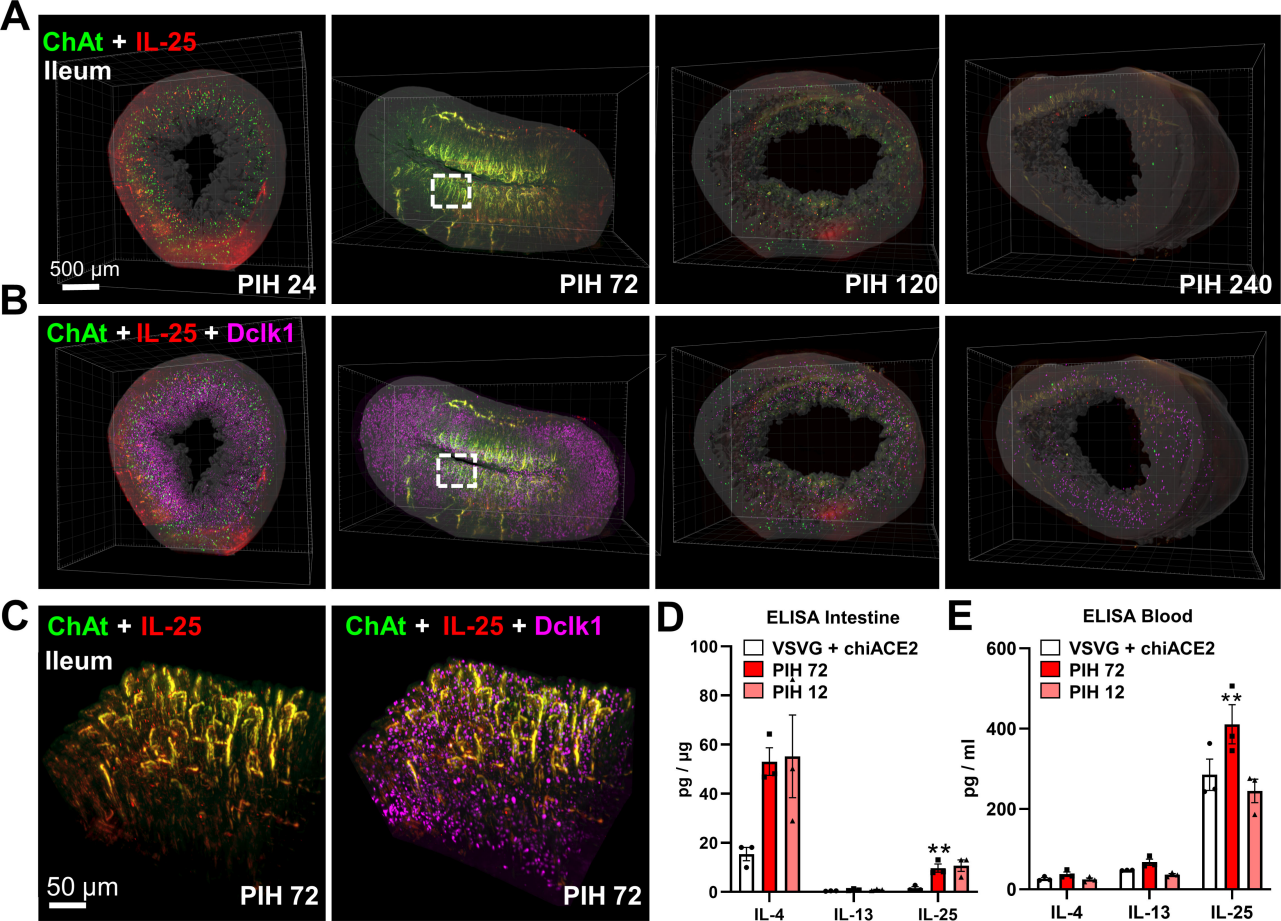
ILC2s correlated with tuft cells and cholinergic innervations in the ileum of the COVID-19-like mouse model. (**A, B**) Representative spatiotemporal changes in cholinergic nerves, tuft cells, and IL-25 in the ileum from PIH 24 to PIH 240, with the second row showing the overlay of Dclk1 on ChAt and IL-25. (**C**) The magnified squares show that ChAt accumulated in the autonomic nerves and that IL-25 surrounded the tuft cells at PIH 72 but became dispersed at later times. (**D, E**) ELISA quantification of the type 2 cytokines, including IL-4, IL-13, and IL-25, in ileum tissue and blood. Data are shown as means ± SEM, *n* = 3/group, *P < 0.05, **P < 0.01, and ***P < 0.001.

By utilizing the enhanced spatial resolution offered by 3D imaging, we successfully reconstructed the cholinergic nerves, tuft cells, and the signaling molecule IL-25 in both lung and ileum tissues (Fig. 6 and 7; Videos S3 and S4). Notably, a significant increase in tuft cells within the mucosal epithelium was observed during PIH 24–72 in both tissues. Furthermore, IL-25 was found to be present and dispersed around the tuft cells and cholinergic nerves (Fig. 6A and 7A). Additionally, ChAt accumulated in neighboring cholinergic nerves at PIH 72, forming continuous, uninterrupted fiber-shaped twists that extended across the tuft cell clusters (Fig. 6B, 7B and C). In the lung, we observed an elevation in tuft cells, cholinergic nerves, and the IL-25 signal (Fig. 6C). Notably, tuft cells exhibited a quantitative increase and were distributed throughout the entire airway during PIH 24–72 (Fig. 6D). Similarly, similar observations were made in the ileum, where tuft cells, cholinergic nerves, and the IL-25 signal were elevated. This was accompanied by increased levels of the type 2 cytokines IL-4 and IL-13 (Fig. 7D). Furthermore, the serum profile indicated a predominance of IL-4, which signifies a type 2 immune response (Fig. 7E). In summary, our findings demonstrate that during the early phase of PSV-S infection, mucosal type 2 immunity expands to involve tuft cells and cholinergic nerves, as evidenced by the increased presence of tuft cells, cholinergic nerves, IL-25, and type 2 cytokines.

Moreover, in the treated and pre-treated chiACE2 mouse model infected with SARS-CoV-2, using unpublished antibodies (Fig. S13), we observed an intensified activity of cholinergic nerves, as well as an increased presence of tuft and goblet cells and IL-25. These compelling findings also indicate the substantial participation of type 2 immunity during the early phases of infection, serving as the primary local defense against the invasion of SARS-CoV-2 in the mucosa. Thus, these results underscore the potential role of tuft cells and cholinergic regulation in this intricate defense mechanism.

## DISCUSSION

SARS-CoV-2 has now lasted for 3 years, and efforts have been made to explore its virulence and how to protect against it. Various animal models have been developed as research tools, among which humanized-ACE2 mice have the advantages of viral susceptibility and similar lung pathology (41). The protein S is sufficient to cause fever and inflammatory lung injury (42). PSV-S has been widely used in the study of COVID-19, such as in neutralization antibody assays, virus assembly, and vaccine development (43), considering its involvement in receptor recognition, viral attachment, and cell entry. Several studies have also reported the pathogenicity of spike protein alone; for example, Yuan et al. administered a pseudovirus-expressed spike protein to Syrian hamsters intratracheally and found spike alone could cause apparent lung damage and severe vascular endothelial cell damage (44). Our aged COVID-19 model used chiACE2 transgenic mice intranasally infected with PSV-S, and these mice were characterized by fever and multi-organ pathology that mimic clinical symptoms in both male and female mice (45). In addition to the obvious lung-gut invasion, the kidney also exhibited severe pathological changes consistent with its abundant ACE2 expression, and this indicated that the kidney is a direct target for organ damage (46). The negative control VSVG pseudovirus-infected chiACE2 model did not show any symptoms or pathological changes, and our experiment using authentic SARS-CoV-2 virus supported the similarity between the pseudovirus and the authentic virus. In addition, the safety of the pseudovirus was indicated by the fact that none of the mice died and all of them self-recovered within 10 days. The last thing worth considering was age, and we used the aged chiACE2 mouse (8 months) while others have only used young mice (around 8 weeks) (47, 48). The underlying mechanisms for why spike protein causes such clinical pathogenesis remain to be further explored. Moreover, mucosal damage, which is one of the most important pathological changes, was found to be common among different organs (49).

Focusing on the lung and intestinal mucosal systems, spatiotemporal interactions between ACE2 and PSV-S were mainly found in the mucosa of the bronchial epithelium and the villi and crypts, demonstrating an important role for the mucosa. Notably, PSV-S was not strictly restricted to ACE2, indicating a role for other potentially necessary (co-)receptors or proteases such as ANPEP and TMPRSS2 (31). Regarding the disparity in colocalization between SARS-CoV-2 and PSV-S with chiACE2, we hypothesize that the variation in spike proteins might be influencing this phenomenon. Additionally, previous reports have indicated that novel coronavirus-Shanghai 01 (nCoV-SH01) (50) induces cytopathic effects on Vero E6 cells at an earlier stage when compared with the previously reported 2019 novel coronavirus (2019-nCoV) (1, 51). This suggests that the differences in spike sequences may contribute to diverse effects on cellular interactions, warranting further investigation. The emergence of the Omicron variant in late November 2021 marked a significant turning point in the pandemic, with successive sub-lineages gaining global dominance (52). Distinguishing itself from previous variants, Omicron displayed lower replication capacity in human lung tissues and utilized a distinct cellular entry mechanism, potentially contributing to its comparatively milder disease severity (53, 54). The future evolutionary trajectory of SARS-CoV-2 remains uncertain, as it is unclear whether the virus will continue to evolve through highly distinct lineages or undergo a more gradual adaptive process (55). To further elucidate the transmission patterns of Omicron, additional research is necessary, building upon the current findings primarily derived from the Alpha variant.

Upon entering cells through ACE2, the primary mucosal immune system is activated and ILC2s appear in an early phase followed by ILC3s. ILC-2 has a protective role against infection (25, 56). Our results highlight the significant role of ILC2s and type 2 immunity in the mucosal crosstalk between the lung and intestine through the simultaneous correlation with tuft cells and cholinergic innervation. The role of ILC2 in COVID-19 is not well understood. Some studies have found a reduction in ILC2 cells associated with more severe disease (25), while others suggest that the presence of ILC2 cells may provide some protection from more severe acute pneumonitis (26). Besides, we have observed higher levels of total ILC1s in the lungs, intestine, and skin. It is worth noting that Marina García et al. also similarly reported a slightly increased percentage of CCR4+ ILC1 cells in COVID-19 patients but no changes in the expression of markers associated with differentiation and activation in ILC1s (25). Despite the limited evidence of direct interactions between ILC2 and ACE2, it is worth noting that ACE2 was found to be expressed at a low level, while TMPRSS2 was reported to be relatively higher in human innate lymphoid cells (57). This higher expression of TMPRSS2 in ILCs could potentially facilitate virus entry into these cells. Another important finding is that ILCs play a significant role in preventing SARS-CoV-2 from entering the endothelial lining by stimulating the production of human defensin-5 (58, 59). Thereafter, further exploration is needed to investigate the direct interaction between ILC2 and ACE2. Our experimental findings suggest that ILC2s may have a protective role in COVID-19, although further research is needed to confirm this. One study suggested that circulating ILC2 cells that express CCR10 may play a role in recovery from acute infection (60). Another study found that a subset of ILC2 cells that express NKG2D was elevated in patients with COVID-19 and negatively correlated with disease severity, suggesting a protective role (28). Overall, more research is needed to understand the role of ILC2 in COVID-19.

Tuft cells are considered to have a Th2-related gene expression signature, with inputs consisting of succinate and taste ligands and outputs consisting of acetylcholine, IL-25, and eicosanoids, thereby regulating type 2 immunity (39, 61). Unlike in the case of allergies or parasites, tuft cells are increased in the early stages of infection to defend against the PSV-S virus under the condition of cholinergic excitation. GO and KEGG analyses indicated the importance of tuft cells in defending against SARS-CoV-2 and other viruses (Fig. 5C and D). Although tuft cells are not the sole source of IL-25, their associated type 2 immunity was found to be part of early mucosal defense mechanisms, which expands our knowledge of the primary role of these cells in mucosal repair and regeneration (18, 29).

Out of a concern for biological safety, this study was limited to a small proportion of samples of SARS-CoV-2 cases. However, accompanied with other 3D spatial or scRNA-seq studies using COVID-19 clinical samples, our data could help provide more information about the mucosal immune landscape and may support other studies investigating systemic or individual organs’ responses to SARS-CoV-2 invasion (62, 63). Another limiting aspect to the current study is the lack of treatment experiments targeted at intensifying the mucosal type 2 immunity or ILC2s, thus making it difficult to establish a causal relationship between ILC2s and goblet cells or tuft cells. Besides, it is necessary to test ILCs and other immunocytes together to demonstrate the significance of ILCs in the immune response induced by SARS-CoV-2. Further experiments are needed to verify the direct causation between the tuft cell-ILC2 axis and SARS-CoV-2.

Broadly speaking, the mucosal system serves as the vital interface connecting various organs with the external environment. Achieving synchronization within this system through interactions involving the nervous, immune, and resident cells necessitates additional investigation. By delving into these complex dynamics, we can gain new insights into systemic physiology and dysfunctions, offering a fresh perspective on the intricate workings of the human body.

### Conclusion

The spike of SARS-CoV-2 binds to ACE2, which enables the PSV-S to break through the mucosal barrier and broadly diffuse into the alveoli or crypts within 72 hours. ILC2s and type 2 immunity function as the primary defense, together with increases in co-related tuft cells and cholinergic innervations. Thus, our results highlight the commonality of mucosal defenses between the lung and gut, particularly type 2 immunity in viral infections (Fig. S14), which underlies the basis of mucosal vaccines and other pre- or post-infection treatment methods for COVID-19.

## MATERIALS AND METHODS

### Reagents and antibodies

Detailed information on the reagents, antibodies, equipment, and software used in the experiments is listed in Table S2.

### Animals

chiACE2 transgenic mice were obtained from Cyagen Biosciences Inc., Guangzhou, China, and C57BL/6J mice were purchased from the Shanghai Laboratory Animal Center (SLAC), Shanghai, China. All of these mice were housed in a barrier facility with a 12-hour light/12-hour dark cycle and humidity of 34%–40% and temperature of 21°C ~ 23°C. Mice were fed ad libitum with the same standard laboratory chow (SLAC, Shanghai, China) and given free access to water. When performing the control or infection model, a single dose was administrated intranasally [5 µL/nostril of 2 × 10^7^ TU/mL for PSV-S or VSVG virus at 8 months of age or 40 µL/nostril, 1 × 10^5^ PFU/mL for nCoV-SH01 strain of SARS-CoV-2 (GenBank accession no. MT121215)], and the day of administration was marked as the starting point. Each mouse was weighed, and their rectal temperature was measured every 12 hours for the first 3 days after infection and then every 24 hours. For each checkpoint, including PIH 24, PIH 72, PIH 120, and PIH 240, six chiACE2 mice were randomly selected and divided equally by sex and sacrificed. The animal experiments were conducted according to the experimental practices, procedures, and standards approved by the Research Ethical Review Committee for Laboratory Animal Welfare of Fudan University. The SARS-CoV-2 infection experiment was conducted in accordance with approved guidelines at the ABSL-3 Lab of Fudan University. The nCoV-SH01 strain of SARS-CoV-2 (GenBank accession no. MT121215) used in this study was obtained from Lab stock virus using Vero E6 (64).

### An ACE2 humanized mouse was constructed using CRISPR/Cas9

Humanized ACE2 mice were generated using CRISPR/Cas9 techniques by Cyagen Biosciences. The *Ace2* gene of the mouse (NCBI Reference Sequence: NM_001130513.1) is located in chromosome X, comprising 19 exons. The ATG start codon is present in exon 2, while the TAG stop codon is located in exon 19. Two guide RNA (gRNA) sequences were used for targeting the reverse strand of the gene: gRNA1 - 5′-CTTGGCATTTTCCTCGGTGAGGG-3′, and gRNA2 - 5′-ATGACAAGTGCTTTGCTAAAAGG-3′.

In brief, the mouse *Ace2* gene was targeted using gRNA, along with a donor vector containing the “ACE2 chimera CDS (human extracellular-mouse helical and cytoplasmic domain)-P2A-EGFP” cassette and Cas9 mRNA. These components were co-injected into fertilized mouse eggs to generate offspring with targeted knock-in mutations. The F0 founder animals were identified through PCR and subsequent sequence analysis. Subsequently, they were bred with wild-type mice to confirm germline transmission and generate F1 animals (Fig. S1).

For the knock-in (KI) model, the region spanning from amino acid 18 to amino acid 805 of the mouse *Ace2* gene was replaced with the “ACE2 chimera CDS (human extracellular-mouse helical and cytoplasmic domain)-P2A linker-EGFP” cassette, resulting in the separate expression of chimera ACE2 and EGFP proteins. Human ACE2 is expressed under the control of the mouse promoter. Additionally, the native murine signal peptide (amino acids 1–17) was retained. The genotyping of the pups will be performed using PCR, followed by sequencing analysis.

### Construction of pseudovirus expressing spike

The pseudovirus expressing the SARS-CoV-2 spike protein was generated using a HIV-based lentivirus system. The construction process involved transient transfection of three plasmids: pMD2.G-SARS-COV-2 Spike envelope, which expresses the spike protein (NC_045512.2) from the Wuhan strain (hu-1) instead of the VSV-G protein (derived from Vesicular stomatitis virus), psPAX2 packaging plasmid, and GPLVX-CMV-tdTomato-T2A-LUC.

To briefly summarize the procedure, HEK-293S cells were transfected with the plasmids, and after 10–12 hours, an enhancing buffer was added, followed by medium replacement after 8 hours. The cells were then cultured for an additional 48 hours, and the cell supernatant, enriched with lentivirus particles, was collected. The lentivirus particles expressing the spike protein (referred to as PSV-S) were concentrated to obtain a high-titer lentivirus concentrate, which was stored at −80°C. Successful transduction of PSV-S was confirmed using HEK293-ACE2 cells *in vitro*.

In addition, a negative control virus was constructed using the pMD2.G plasmid to express the envelope protein VSVG, along with the psPAX2 and GPLVX-CMV-tdTomato-T2A-LUC plasmids.

### Quantitative real-time PCR analysis

Total RNA was extracted from serum using a Viral RNA Mini Kit (QIAamp, QIAGEN, China), and cDNA was synthesized using an iScript cDNA Synthesis Kit (BIO-RAD Inc., USA) following the manufacturer’s protocol. Quantitative real-time PCR analysis was performed using the SYBR Premix Ex Taq Kit (Cat# RR420A, Takara Bio Inc.) and specific primers (Table S3) using the ABI PRISM 7900 sequencing system (Applied Biosystems, Foster, CA). All experiments were performed in triplicate, and the mean was used to reduce measurement error.

### Cytokine analysis

Serum and intestine tissues were collected for the ELISA test. Intestines were collected from mice and washed free of feces with PBS, and 50 mg of intestine tissue was dissected and lysed in RIPA lysis buffer containing Protease Inhibitor Cocktail (Cat# PCDBE101, P&C Biotechnology Ltd., China). The tissues were fully ground (4°C, 30 min) and centrifuged (15,000 × *g*, 4°C, 30 min) to obtain the supernatant, and a BCA kit (Cat# YSD-500T, Yoche Biotechnology Ltd., China) was used to quantify the total protein content. The ELISA was standardized using the BCA results. Serum was obtained from blood samples centrifuged at 3,000 rpm for 20 min, and the protein content was similarly calculated by BCA. The intestinal supernatant was diluted threefold, and serum was used at its original concentration. Cytokine levels (including IL-4, IL-13, and IL-25) in tissue homogenates or serum were measured using ELISA kits (ELISA kit PCDBM0168 for IL-4, PCDBM0147 for IL-13, and PCDBM0130 for IL-25, P&C Biotechnology Ltd., China) with a STAT FAX 2100 Microplate Reader (Awareness Technology Inc., USA).

### Multicolor flow cytometry

Cells from the skin, lung, and intestines were isolated and stained for multicolor flow cytometry as previously reported (65 – 67). In brief, bilateral ears, thoracic and abdominal skins, middle and inferior lobes of the right lungs, and the whole intestines were collected for isolation of skin, lung, and intestinal cells, respectively. All collected tissues were cut into 2 mm × 2 mm × 2 mm pieces and subjected to both physical and chemical digestion (37°C). The lung tissue was digested in RPMI-1640 medium supplemented with 50 µg/mL Liberase and 1 µg/mL Dnase 1. The intestine tissue was pre-digested with 1× HBSS supplemented with 10 mM HEPES, 5 mM EDTA, 1 mM DTT, and 5% FBS and digested with RPMI-1640 supplemented with 80 UI/mL Dnase 1, 3.94 UI/mL Dispase II, and 10% FBS. The skin was digested with RPMI-1640 supplemented with 0.75 mg/mL collagenase I and 0.3 mg/mL Dnase I. The digested solutions were filtered through 70 µM cell filters to obtain a single-cell solution, and isolated cells were analyzed by flow cytometry using the antibodies listed in Table S2. A lineage cocktail containing monoclonal antibodies to B220, CD3, CD11c, CD16/32, and CD11b was used except where stated, and ILCs were identified as IL-7Rα+/Lin– cells. Surface staining was performed with antibodies diluted in DPBS supplemented with 2% FBS and 0.5% EDTA at 4°C for 30 min. All cells were treated with Fc-receptor block. For the intracellular staining, cells were harvested after centrifuging at 350 × *g* for 5 min. After fixing and permeabilizing using the Foxp3 Staining Buffer Set (eBioscience, Cat# 00–5523-00, USA) according to the manufacturer’s instructions, intracellular T-bet, GATA3, and RORγt were stained by adding primary antibodies. Samples were run on a BD LSRFortessa TM X-20 (BD Biosciences), and data were collected using the BD FACSDiva Software (BD Biosciences).

### Immunofluorescence staining

Cryosections were obtained after 30% sucrose dehydration and were stained following traditional immunostaining methods. Briefly, sections underwent autofluorescence quenching (Vector TrueVIEW Autofluorescence Quenching Kit SP-8400, Hongkong), antigen retrieval and blocking (41 mL 0.1 M sodium citrate and 9 mL 0.1 M citrate in 500 mL boiling ddH2O twice for retrieval followed by 0.01% PBST, 5% BSA, and 5% donkey serum for blocking), and primary antibody reaction with the appropriate secondary antibodies (Table S2). Images were obtained with a fluorescence microscope (NE900-FL, Ningbo Yongxin Optics Co. Ltd.).

### HE and AB-PAS staining

HE and AB-PAS staining were performed according to the manufacturer’s protocol (Hematoxylin-Eosin Staining Kit (G1120), Alcian Blue Periodic-acid Schiff Stain Kit (G1285), Solarbio Life Science, Peking). HE staining of the ileum and lung were judged by a qualified pathologist under double-blind conditions and scored according to the published criteria (68, 69).

### Re-analysis of public scRNA-Seq data from COVID-19 patients

The published scRNA-seq data were downloaded from the NCBI GEO database under the accession number GSE171524. The scRNA-seq expression data were analyzed with Seurat (PCA, Cluster, and t-SNE). Briefly, Seurat objects were generated from digital gene expression matrices. Nineteen principal components were used in the cell cluster with the resolution parameter set at 0.5, and the vloplot function was used to visualize the expression levels of the *IL25*, *IL33*, and *DCLK1* genes in each cell cluster. The dotplot function was used to visualize the expression levels of tuft cell-related signaling markers in major pulmonary cell types. GO and KEGG enrichment analysis of identified marker genes for each cell cluster was performed using clusterProfiler.

### iDISCO tissue transparency method

The iDISCO method was performed according to the protocol of Renier et al. (70). Briefly, mice were perfused intracardially with 20 mL 1× phosphate-buffered saline (4°C, containing 10 U/mL heparin) to wash out the blood, followed by 4% paraformaldehyde embedding at 4°C for 3 days. The right caudal lobe of the lung and a 5-mm section of the ileum were dissected for iDISCO clearing. Before clearing, samples were pre-treated with autofluorescence quenching for 1 day and blocking solution for 3 days at 37°C on a shaker. The samples were then dehydrated in a series of methanol in PBS (20%, 40%, 60%, 80%, and 100%, one hour each at room temperature) and incubated with 66% DCM/33% methanol at 4°C overnight. Rehydration by reversing the methanol gradient was then performed before immunolabeling, followed by permeabilization, blocking, and washing with PTx.2 to enable deeper penetration of antibodies and to decrease nonspecific binding. For immunostaining, primary antibody incubation was followed by secondary antibody incubation at 37°C for 3 days (Table S2), and PTwH was used to wash out any unbound antibodies. The final step was reflex index calibration, and the samples were immersed in dibenzyl ether until clear (usually overnight) to prepare them for the 3D imaging.

### Lightsheet 3D imaging and image analysis

Three-dimensional fluorescence imaging was performed using a lightsheet microscope (LS-18, Nuohai Co. Ltd.), and Imaris software (version 9.8, Oxford Instruments PLC, UK) was used for the 3D rendering and quantitative analysis. Lightsheet imaging was performed based on the protocol of Gao et al. (71). Briefly, a 6.3×/0.25 NA lens was used, with bilateral illumination of three channels excited at 488 nm, 594 nm, and 647 nm. Exposure time (50 ms) and power (150 mW) were adjusted consistently for all the samples, and images were obtained with an ORCA Flash 4.0 camera with a resolution of 3.2 µM in the x and y axes and 7 µM in the z axis. For the tile scanning model, the imaging was tiled three times to obtain the global focus.

“Imaging processing” and “3D” Crop were used to display the panoramic view with the better resolution. Several algorithms were used for collecting quantitative statistics, including *Surface* and *Xtensions*, after which the spatial parameters could be automatically calculated.

The quantitative statistical analysis is comprised of three main steps: segmentation, reconstruction, and statistical analysis (72). Firstly, for the intestine, villi and crypts were classified based on their morphology and distance from the mucosa. For the lung, the branch hierarchy was based on the bronchi diameter, and the nomenclature followed established rules (73). Then, “Surface” was reconstructed for the region of interest and analyzed for the 3D volume of positive signal, as well as the 3D coordinate. The volume of crypt and villa in the intestine, airways in each lung segment, hACE2, spike, Dclk1, ChAt, and IL-25 were calculated. The entrance of spike pseudovirus, depending on hACE2, was also calculated based on the 3D coordinate using *Xtensions* (73).

### Statistical analysis

All statistical analyses were performed with GraphPad Prism v8.0.2 (La Jolla, CA, USA) and presented as the means ± standard error of the mean in the figures. The Shapiro-Wilk test was first used to verify the normal distribution of all the data sets, and the differences among groups were assessed with one-way analysis of variance (ANOVA) followed by Dunnett’s multiple comparison tests and two-tailed Student’s (*t*-test). *P < 0.05 and **P < 0.01 were considered significant.

## Data Availability

Data available within the article or its supplementary materials.

## SUPPLEMENTAL MATERIAL

Tables S1 to S4 spectrum.00878-23-s0001.docx Supplementary tables.

Legends to Videos S1 to S4 spectrum.00878-23-s0002.docx Supplementary video legends.

Fig. S1 to S14 spectrum.00878-23-s0003.pdf Supplementary figures and legends.

Video S1 spectrum.00878-23-s0004.mp4 3D reconstruction of spike and chiACE2 in the lungs of the COVID-19-like mouse model.

Video S2 spectrum.00878-23-s0005.mp4 3D reconstruction of spike and chiACE2 in the ileum of the COVID-19-like mouse model.

Video S3 spectrum.00878-23-s0006.mp4 3D reconstruction of cholinergic nerves (ChAt), IL-25, and tuft cells (Dclk1) in the lungs of the COVID-19-like mouse model.

Video S4 spectrum.00878-23-s0007.mp4 3D reconstruction of cholinergic nerves (ChAt), IL-25, and tuft cells (Dclk1) in the ileum of the COVID-19-like mouse model.

ASM does not own the copyrights to Supplemental Material that may be linked to, or accessed through, an article. The authors have granted ASM a non-exclusive, world-wide license to publish the Supplemental Material files. Please contact the corresponding author directly for reuse.
