# Supplementary material for: Spatiotemporal observations of host-pathogen interactions in mucosa during SARS-CoV-2 infection indicate a protective role of ILC2s
Source: Microbiol Spectr. 2023 Nov 8;11(6):e00878-23. doi: 10.1128/spectrum.00878-23 (PMC10714800; doi:10.1128/spectrum.00878-23)
Supplement: Tables S1 to S4 — Supplementary tables. [file spectrum.00878-23-s0001.docx]

**Supplementary table 1. Amino acids and CDS sequence**

Amino acids sequence

Mouse Ace2 sequence、Human ACE2 sequence、P2A、EGFP

1 MSSSSWLLLS LVAVTTAQST IEEQAKTFLD KFNHEAEDLF YQSSLASWNY

51 NTNITEENVQ NMNNAGDKWS AFLKEQSTLA QMYPLQEIQN LTVKLQLQAL

101 QQNGSSVLSE DKSKRLNTIL NTMSTIYSTG KVCNPDNPQE CLLLEPGLNE

151 IMANSLDYNE RLWAWESWRS EVGKQLRPLY EEYVVLKNEM ARANHYEDYG

201 DYWRGDYEVN GVDGYDYSRG QLIEDVEHTF EEIKPLYEHL HAYVRAKLMN

251 AYPSYISPIG CLPAHLLGDM WGRFWTNLYS LTVPFGQKPN IDVTDAMVDQ

301 AWDAQRIFKE AEKFFVSVGL PNMTQGFWEN SMLTDPGNVQ KAVCHPTAWD

351 LGKGDFRILM CTKVTMDDFL TAHHEMGHIQ YDMAYAAQPF LLRNGANEGF

401 HEAVGEIMSL SAATPKHLKS IGLLSPDFQE DNETEINFLL KQALTIVGTL

451 PFTYMLEKWR WMVFKGEIPK DQWMKKWWEM KREIVGVVEP VPHDETYCDP

501 ASLFHVSNDY SFIRYYTRTL YQFQFQEALC QAAKHEGPLH KCDISNSTEA

551 GQKLFNMLRL GKSEPWTLAL ENVVGAKNMN VRPLLNYFEP LFTWLKDQNK

601 NSFVGWSTDW SPYADQSIKV RISLKSALGD KAYEWNDNEM YLFRSSVAYA

651 MRQYFLKVKN QMILFGEEDV RVANLKPRIS FNFFVTAPKN VSDIIPRTEV

701 EKAIRMSRSR INDAFRLNDN SLEFLGIQPT LGPPNQPPVS IWLIIFGVVM

751 ALVVVGIIIL IVTGIKGRKK KNETKREENP YDSMDIGKGE SNAGFQNSDD

801 AQTSFGSGAT NFSLLKQAGD VEENPGPMVS KGEELFTGVV PILVELDGDV

851 NGHKFSVSGE GEGDATYGKL TLKFICTTGK LPVPWPTLVT TLTYGVQCFS

901 RYPDHMKQHD FFKSAMPEGY VQERTIFFKD DGNYKTRAEV KFEGDTLVNR

951 IELKGIDFKE DGNILGHKLE YNYNSHNVYI MADKQKNGIK VNFKIRHNIE

1001 DGSVQLADHY QQNTPIGDGP VLLPDNHYLS TQSALSKDPN EKRDHMVLLE

1051 FVTAAGITLG MDELYK*

CDS

Mouse Ace2 sequence、Human ACE2 sequence、P2A、EGFP

1 ATGTCCAGCT CCTCCTGGCT CCTTCTCAGC CTTGTTGCTG TTACTACTGC TCAGTCCACC

61 ATTGAGGAAC AGGCCAAGAC ATTTTTGGAC AAGTTTAACC ACGAAGCCGA AGACCTGTTC

121 TATCAAAGTT CACTTGCTTC TTGGAATTAT AACACCAATA TTACTGAAGA GAATGTCCAA

181 AACATGAATA ATGCTGGGGA CAAATGGTCT GCCTTTTTAA AGGAACAGTC CACACTTGCC

241 CAAATGTATC CACTACAAGA AATTCAGAAT CTCACAGTCA AGCTTCAGCT GCAGGCTCTT

301 CAGCAAAATG GGTCTTCAGT GCTCTCAGAA GACAAGAGCA AACGGTTGAA CACAATTCTA

361 AATACAATGA GCACCATCTA CAGTACTGGA AAAGTTTGTA ACCCAGATAA TCCACAAGAA

421 TGCTTATTAC TTGAACCAGG TTTGAATGAA ATAATGGCAA ACAGTTTAGA CTACAATGAG

481 AGGCTCTGGG CTTGGGAAAG CTGGAGATCT GAGGTCGGCA AGCAGCTGAG GCCATTATAT

541 GAAGAGTATG TGGTCTTGAA AAATGAGATG GCAAGAGCAA ATCATTATGA GGACTATGGG

601 GATTATTGGA GAGGAGACTA TGAAGTAAAT GGGGTAGATG GCTATGACTA CAGCCGCGGC

661 CAGTTGATTG AAGATGTGGA ACATACCTTT GAAGAGATTA AACCATTATA TGAACATCTT

721 CATGCCTATG TGAGGGCAAA GTTGATGAAT GCCTATCCTT CCTATATCAG TCCAATTGGA

781 TGCCTCCCTG CTCATTTGCT TGGTGATATG TGGGGTAGAT TTTGGACAAA TCTGTACTCT

841 TTGACAGTTC CCTTTGGACA GAAACCAAAC ATAGATGTTA CTGATGCAAT GGTGGACCAG

901 GCCTGGGATG CACAGAGAAT ATTCAAGGAG GCCGAGAAGT TCTTTGTATC TGTTGGTCTT

961 CCTAATATGA CTCAAGGATT CTGGGAAAAT TCCATGCTAA CGGACCCAGG AAATGTTCAG

1021 AAAGCAGTCT GCCATCCCAC AGCTTGGGAC CTGGGGAAGG GCGACTTCAG GATCCTTATG

1081 TGCACAAAGG TGACAATGGA CGACTTCCTG ACAGCTCATC ATGAGATGGG GCATATCCAG

1141 TATGATATGG CATATGCTGC ACAACCTTTT CTGCTAAGAA ATGGAGCTAA TGAAGGATTC

1201 CATGAAGCTG TTGGGGAAAT CATGTCACTT TCTGCAGCCA CACCTAAGCA TTTAAAATCC

1261 ATTGGTCTTC TGTCACCCGA TTTTCAAGAA GACAATGAAA CAGAAATAAA CTTCCTGCTC

1321 AAACAAGCAC TCACGATTGT TGGGACTCTG CCATTTACTT ACATGTTAGA GAAGTGGAGG

1381 TGGATGGTCT TTAAAGGGGA AATTCCCAAA GACCAGTGGA TGAAAAAGTG GTGGGAGATG

1441 AAGCGAGAGA TAGTTGGGGT GGTGGAACCT GTGCCCCATG ATGAAACATA CTGTGACCCC

1501 GCATCTCTGT TCCATGTTTC TAATGATTAC TCATTCATTC GATATTACAC AAGGACCCTT

1561 TACCAATTCC AGTTTCAAGA AGCACTTTGT CAAGCAGCTA AACATGAAGG CCCTCTGCAC

1621 AAATGTGACA TCTCAAACTC TACAGAAGCT GGACAGAAAC TGTTCAATAT GCTGAGGCTT

1681 GGAAAATCAG AACCCTGGAC CCTAGCATTG GAAAATGTTG TAGGAGCAAA GAACATGAAT

1741 GTAAGGCCAC TGCTCAACTA CTTTGAGCCC TTATTTACCT GGCTGAAAGA CCAGAACAAG

1801 AATTCTTTTG TGGGATGGAG TACCGACTGG AGTCCATATG CAGACCAAAG CATCAAAGTG

1861 AGGATAAGCC TAAAATCAGC TCTTGGAGAT AAAGCATATG AATGGAACGA CAATGAAATG

1921 TACCTGTTCC GATCATCTGT TGCATATGCT ATGAGGCAGT ACTTTTTAAA AGTAAAAAAT

1981 CAGATGATTC TTTTTGGGGA GGAGGATGTG CGAGTGGCTA ATTTGAAACC AAGAATCTCC

2041 TTTAATTTCT TTGTCACTGC ACCTAAAAAT GTGTCTGATA TCATTCCTAG AACTGAAGTT

2101 GAAAAGGCCA TCAGGATGTC CCGGAGCCGT ATCAATGATG CTTTCCGTCT GAATGACAAC

2161 AGCCTAGAGT TTCTGGGGAT ACAGCCAACA CTTGGACCTC CTAACCAGCC CCCTGTTTCC

2221 ATATGGCTGA TTATTTTTGG TGTTGTGATG GCACTGGTAG TGGTTGGCAT CATCATCCTG

2281 ATTGTCACTG GGATCAAAGG TCGAAAGAAG AAAAATGAAA CAAAAAGAGA AGAGAACCCT

2341 TATGACTCGA TGGACATTGG AAAAGGAGAA AGCAATGCAG GATTCCAAAA CAGTGATGAT

2401 GCTCAGACTT CCTTTGGAAG CGGAGCCACG AACTTCTCTC TGTTAAAGCA AGCAGGAGAT

2461 GTTGAAGAAA ACCCCGGGCC TATGGTGAGC AAGGGCGAGG AGCTGTTCAC CGGGGTGGTG

2521 CCCATCCTGG TCGAGCTGGA CGGCGACGTA AACGGCCACA AGTTCAGCGT GTCCGGCGAG

2581 GGCGAGGGCG ATGCCACCTA CGGCAAGCTG ACCCTGAAGT TCATCTGCAC CACCGGCAAG

2641 CTGCCCGTGC CCTGGCCCAC CCTCGTGACC ACCCTGACCT ACGGCGTGCA GTGCTTCAGC

2701 CGCTACCCCG ACCACATGAA GCAGCACGAC TTCTTCAAGT CCGCCATGCC CGAAGGCTAC

2761 GTCCAGGAGC GCACCATCTT CTTCAAGGAC GACGGCAACT ACAAGACCCG CGCCGAGGTG

2821 AAGTTCGAGG GCGACACCCT GGTGAACCGC ATCGAGCTGA AGGGCATCGA CTTCAAGGAG

2881 GACGGCAACA TCCTGGGGCA CAAGCTGGAG TACAACTACA ACAGCCACAA CGTCTATATC

2941 ATGGCCGACA AGCAGAAGAA CGGCATCAAG GTGAACTTCA AGATCCGCCA CAACATCGAG

3001 GACGGCAGCG TGCAGCTCGC CGACCACTAC CAGCAGAACA CCCCCATCGG CGACGGCCCC

3061 GTGCTGCTGC CCGACAACCA CTACCTGAGC ACCCAGTCCG CCCTGAGCAA AGACCCCAAC

3121 GAGAAGCGCG ATCACATGGT CCTGCTGGAG TTCGTGACCG CCGCCGGGAT CACTCTCGGC

3181 ATGGACGAGC TGTACAAGTA A

**Supplementary table 2.** **Reagent, antibody, equipment, and software**

| **Name** | **Cat. number** | **Manufacturer** |  |
| --- | --- | --- | --- |
| **Reagent** | | |  |
| Isoflurane | R510-22-16 | Shenzhen RWD Life Science, Co., Ltd., China |  |
| Heparin | M5688 | Macleans, USA |  |
| 4% paraformaldehyde (PFA) | CR10010 | Crystal-bio, China |  |
| PBS | C10010500BT | Gibco, USA |  |
| Methanol | 322415 | Sigma-Aldrich, USA |  |
| DiChloroMethane (DCM) | 270997 | Sigma-Aldrich, USA |  |
| PTx.2 |  |  |  |
| PBS | C10010500BT | Gibco, USA |  |
| Triton X-100 | V900502 | Sigma-Aldrich, USA |  |
| PTwH |  |  |  |
| PBS | C10010500BT | Gibco, USA |  |
| Tween-20 | 28320 | ThermoFisher, USA |  |
| Heparin | M5688 | Macleans, USA |  |
| Permeabilization Solution |  |  |  |
| PTx.2 |  |  |  |
| Glycine | G8898 | Sigma-Aldrich, USA |  |
| DMSO | D9170 | Sigma-Aldrich, USA |  |
| Blocking Solution |  |  |  |
| PTx.2 |  |  |  |
| Donkey serum | D9663 | Sigma-Aldrich, USA |  |
| DMSO | D9170 | Sigma-Aldrich, USA |  |
| Dibenzyl ether | 108014 | Sigma-Aldrich, USA |  |
| **Antibody** | | |  |
| ACE2 | AF933 | Abcam |  |
| RFP | 600-401-379 | Rockland |  |
| Dclk1 | ab31704 | Abcam |  |
| IL25 | NBP1-30052 | NOVUS |  |
| IL33 | PA5-47007 | ThermoFisher |  |
| IL4 | YT2334 | Immunoway |  |
| IL13 | ab108501 | Abcam |  |
| ChAt | NBP1-30052 | NOVUS |  |
| Tuj1 | ab18207 | Abcam |  |
| NeuN | ab177487 | Abcam |  |
| TH | Ab112 | Abcam |  |
| Alexa 405 anti-rabbit | ab175651 | Abcam |  |
| Alexa 647 anti-rabbit | A32733 | ThermoFisher |  |
| Alexa 488 anti-goat | A32814 | ThermoFisher |  |
| Alexa 594 anti-rat | A-21209 | ThermoFisher |  |
| DAPI | D9542 | Sigma-Aldrich |  |
| **Multicolor flow cytometry** | | |  |
| B220 | RA3-6B3 | Biolegend |  |
| CD3 | 17-A2 | Biolegend |  |
| CD11c | N418 | Biolegend |  |
| CD11b | M1/70 | Biolegend |  |
| CD16/32 | 93 | Biolegend |  |
| IL-7Rα+(CD127) | A7R34 | Biolegend |  |
| T-bet | 4B10 | eBioscience |  |
| GATA3 | TWAJ | eBioscience |  |
| RORγt | AFKTJS-9 | eBioscience |  |
| **Apparatus** |  |  |  |
| Florescence microscope | Primo star | Zeiss, German |  |
| Light Sheet microscope | LS-18 | Nuohai Life Science Co., Ltd., China |  |
| Work station | W-2245 | Intel |  |
| Small animal rectal thermometer | LAT-212 | Lab Animal Technology Develop Co., Ltd., China |  |
| **Software** | | |  |
| Imaris | Imaris 9.8 | Bitplane, Switzerland |  |
| GraphPad Prism | version 8.0.2 | GraphPad |  |

**Supplementary table 3. Primers for qRT-PCR**

| **Gene** |  | **Primer (from 5’ to 3’)** |
| --- | --- | --- |
| hACE2 allele primer 1 | F1 | CCTGCTTCCACTCCTTATTAGCCT |
|  | R1 | TGAAACTGGAATTGGTAAAGGGTC |
| hACE2 allele primer 2 | F2 | TGAATAATGCTGGGGACAAATGG |
|  | R2 | GAGGATAGAATTGGTTCTTAGGAAGG |
| hACE2 allele primer 3 | F3 | CAGCAGCTTGTTTACTGTTCTCTTC |
|  | R3 | AAGAGATGTCAAATCCTTAGGCAG |
| WPRE | Forward | CGCTATGTGGATACGCTGCTTTA |
|  | Reverse | GCAACCAGGATTTATACAAGGAGGA |
| Luc | Forward | AATGTCCGTTCGGTTGGCAG |
|  | Reverse | GGCTGCGAAATGCCCATACT |
| Acc1 | Forward | CACATCATGAAGGAGGAGG |
|  | Reverse | GCTATCACACAGCCTGGGTC |

**Supplementary table 4. Hierarchy of lung bronchus**

|  | **Max / μm** | **Min / μm** |
| --- | --- | --- |
| **CaRMB3** | 972.50 ± 4.93 | 916.25 ± 2.29 |
| **CaRMB4** | 914.25 ± 1.57 | 837.50 ± 3.53 |
| **CaRMB5** | 687.00 ± 3.65 | 627.00 ± 2.72 |
| **CaRMB6** | 467.00 ± 3.68 | 418.00 ± 2.16 |
| **CaRMB7** | 274.00 ± 1.35 | 247.75 ± 2.83 |
| **CaRMB8** | 132.00 ± 5.79 | 101.68 ± 1.65 |
