# Supplementary material for: Spatiotemporal observations of host-pathogen interactions in mucosa during SARS-CoV-2 infection indicate a protective role of ILC2s
Source: Microbiol Spectr. 2023 Nov 8;11(6):e00878-23. doi: 10.1128/spectrum.00878-23 (PMC10714800; doi:10.1128/spectrum.00878-23)
Supplement: Legends to Videos S1 to S4 — Supplementary video legends. [file spectrum.00878-23-s0002.docx]

**Supplementary Video 1.**

3D reconstruction of spike and chiACE2 in the lungs of the COVID-19–like mouse model.

**Supplementary Video 2.**

3D reconstruction of spike and chiACE2 in the ileum of the COVID-19–like mouse model.

**Supplementary Video 3.**

3D reconstruction of cholinergic nerves (ChAt), IL-25, and tuft cells (Dclk1) in the lungs of the COVID-19–like mouse model.

**Supplementary Video 4.**

3D reconstruction of cholinergic nerves (ChAt), IL-25, and tuft cells (Dclk1) in the ileum of the COVID-19–like mouse model.
