# Supplementary material for: Spatiotemporal observations of host-pathogen interactions in mucosa during SARS-CoV-2 infection indicate a protective role of ILC2s
Source: Microbiol Spectr. 2023 Nov 8;11(6):e00878-23. doi: 10.1128/spectrum.00878-23 (PMC10714800; doi:10.1128/spectrum.00878-23)
Supplement: Fig. S1 to S14 — Supplementary figures and legends. [file spectrum.00878-23-s0003.pdf]

Hu et al. Supplementary Figure 1

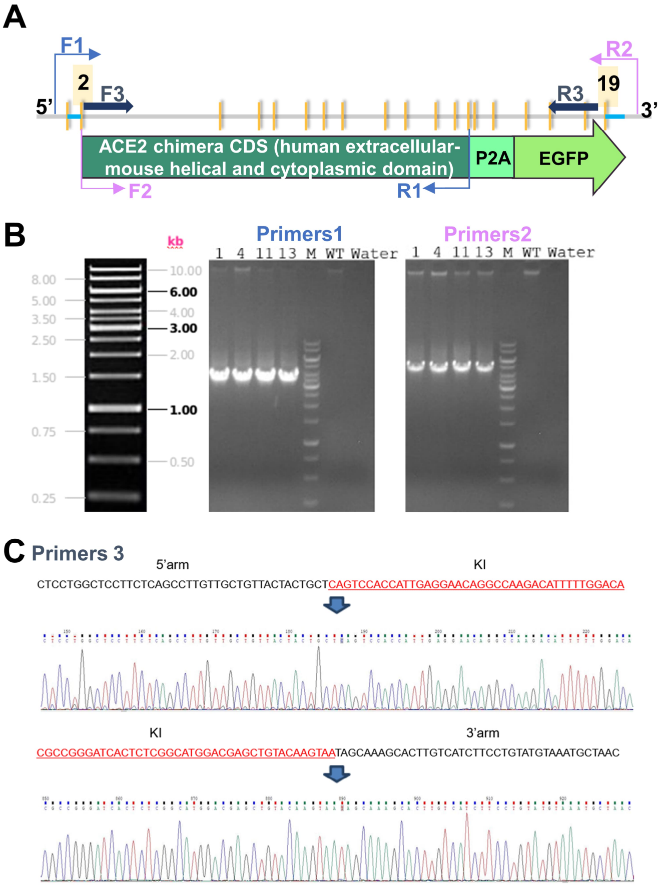

Supplementary Figure 1. Construction of the chiACE2 transgenic mouse.
(A) Schematic diagram for the chiACE2 transgenic strategy with the testing site. **(B)** and **(C)** PCR results showing the accuracy of chiACE2 CDS replacement. KI, knockin.

Hu et al. Supplementary Figure 2

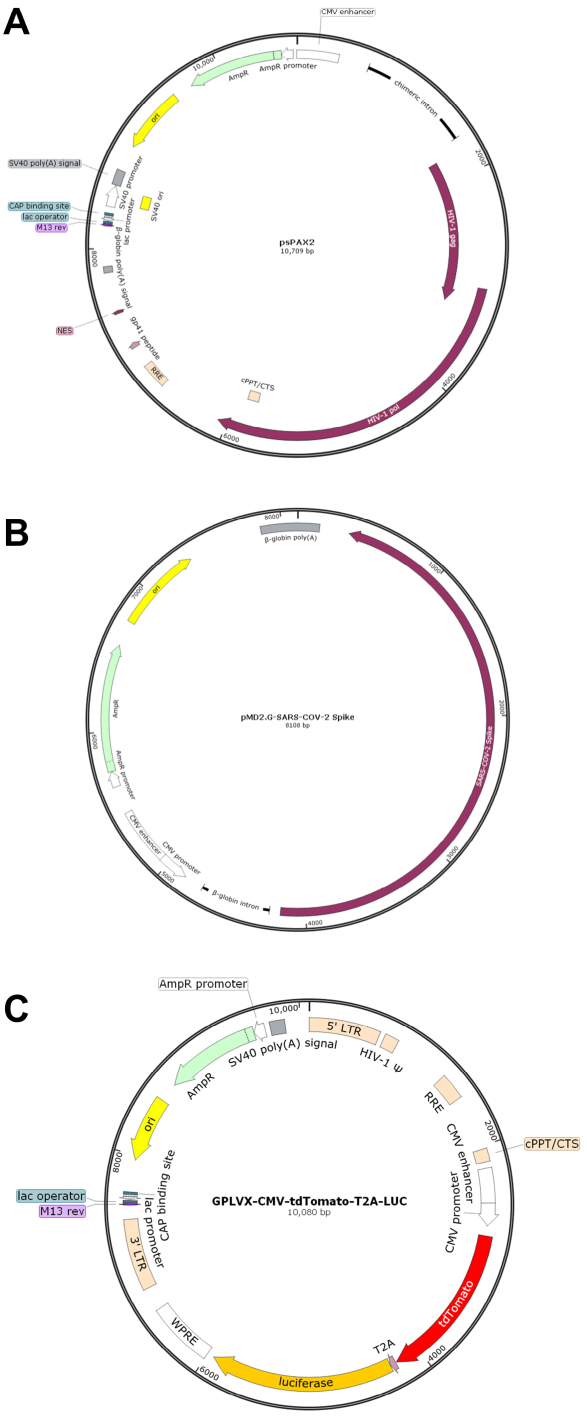

Supplementary Figure 2. Plasmid for Construction of PSV-S

(A) psPAX2. **(B)** pMD2.G-SARS-COV-2 spike. **(C)** GPLVX-CMV-tdTomato-T2A-LUC

Hu et al. Supplementary Figure 3

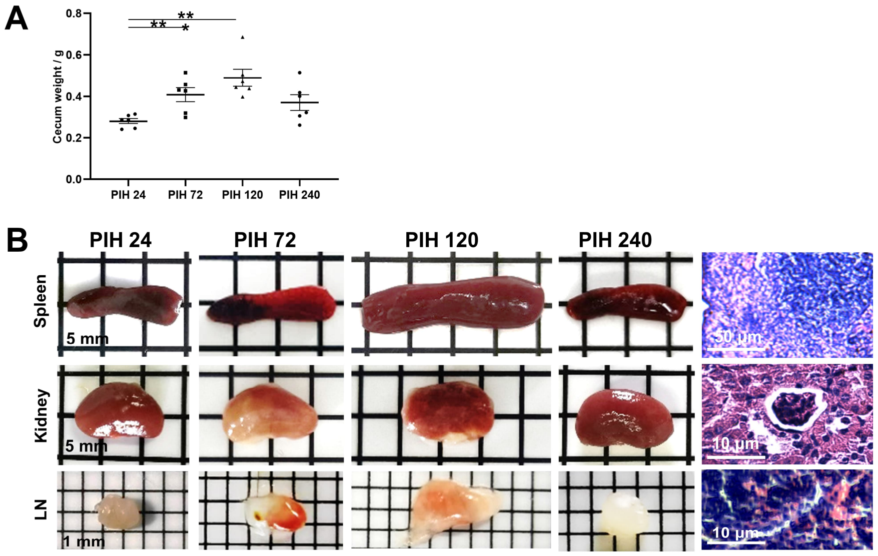

Supplementary Figure 3. The COVID-19–like chiACE2 mouse model exhibited multi-organ dysfunction

(A) Caecum weight change with the time after infection. **(B-D)** Visible gross and pathological changes in the spleen, kidney, and lymph nodes (LN).

Hu et al. Supplementary Figure 4

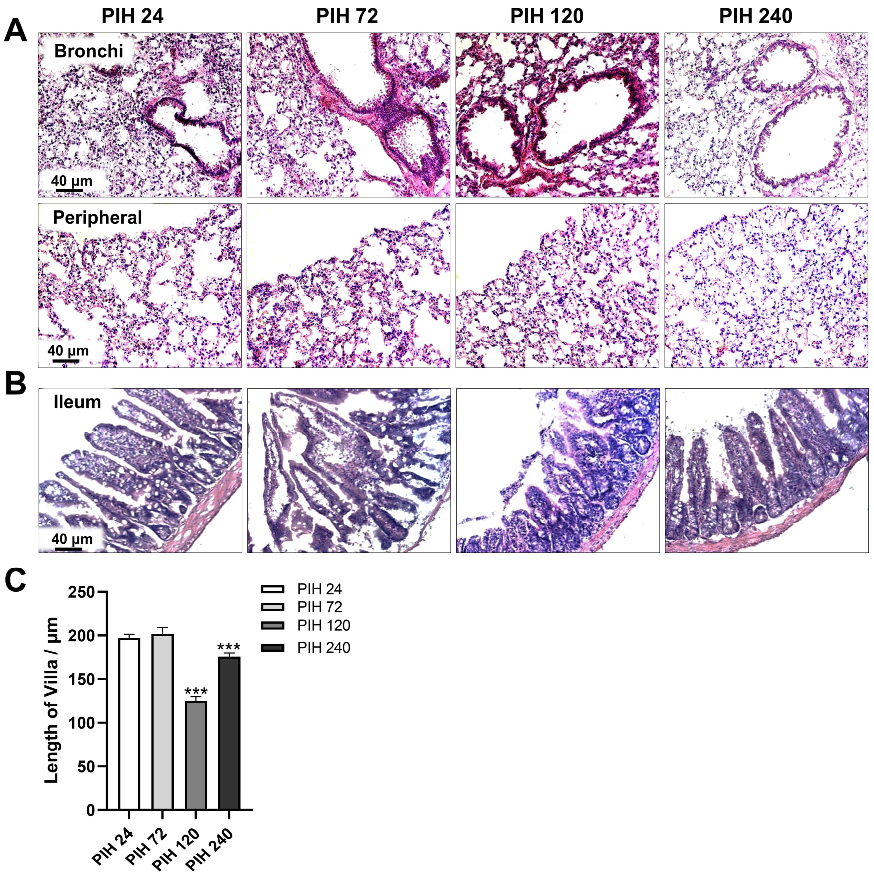

Supplementary Figure 4. HE staining of the lung and ileum of the COVID-19–like mouse model.

(A) and (B) display representative staining utilized for the calculation of the HE score. **(C)** presents the statistical analysis regarding the shortened villa.

Hu et al. Supplementary Figure 5

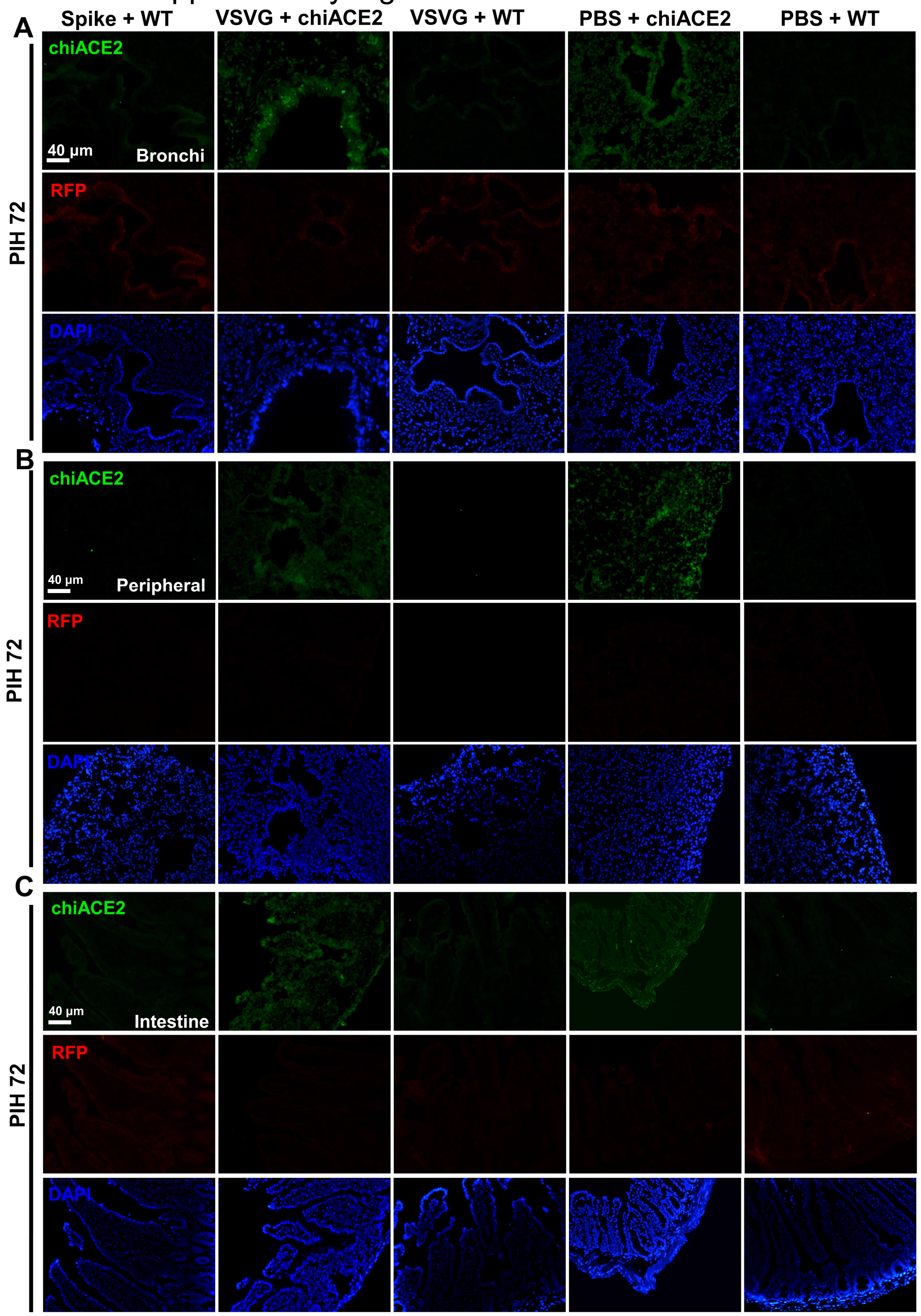

Supplementary Figure 5. Negative control for chiACE2 intranasal infection with the PSV-S model.

VSVG or PBS served as the negative control for PSV-S, and WT mice were the negative control for chiACE2 mice. **(A)**, **(B)**, and **(C)** Representative images of the lung bronchus, peripheral alveolus, and intestine.

Hu et al. Supplementary Figure 6

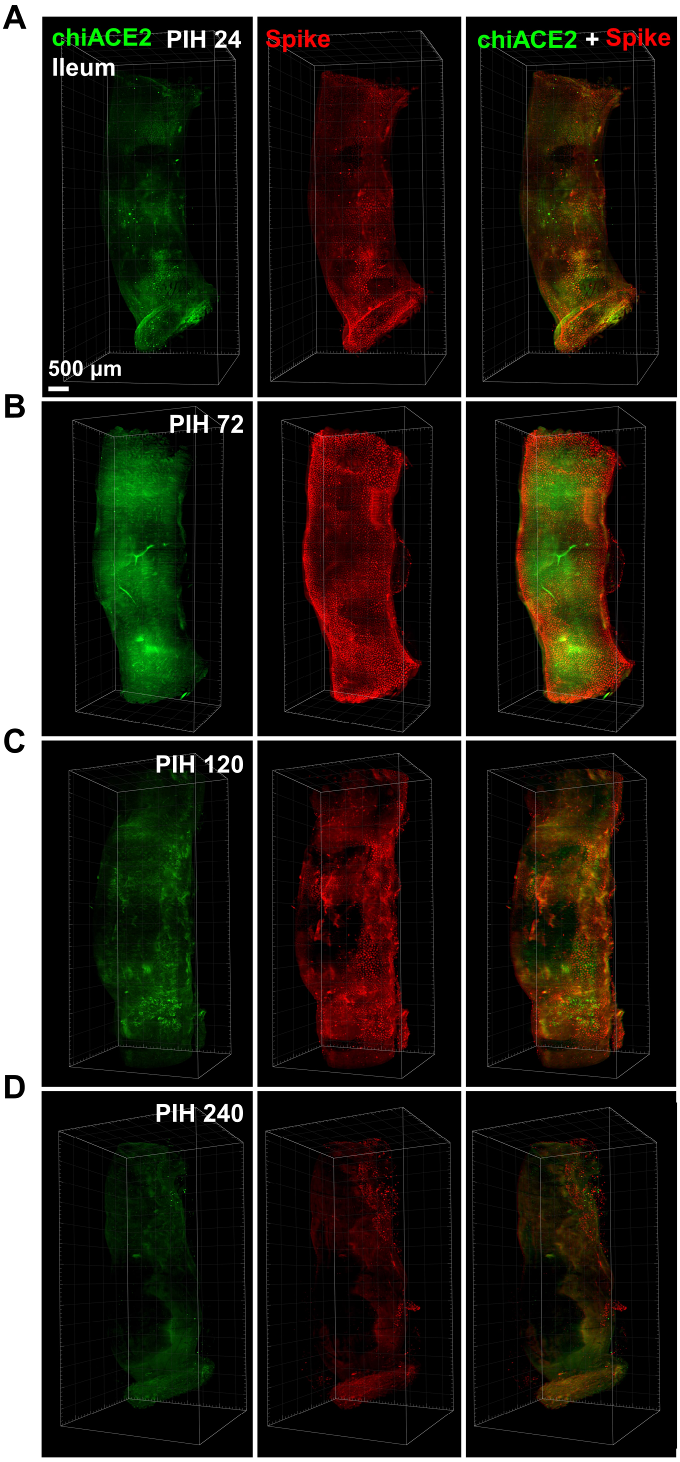

Supplementary Figure 6. 3D view of chiACE2 and PSV-S showing the spatiotemporal pattern in the ileum of the COVID-19–like mouse model.
Induced chiACE2 bound to PSV-S reached its maximum at PIH 72.

Hu et al. Supplementary Figure 7

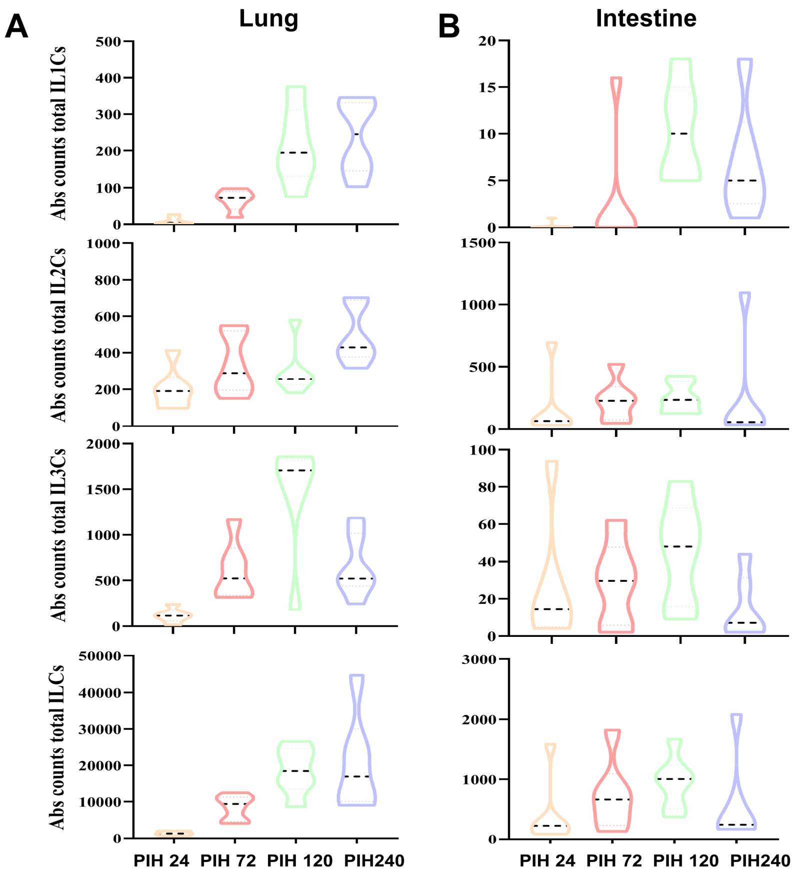

Supplementary Figure 7. Post-infection changes in ILCs in the pulmonary and intestinal mucosa of the COVID-19–like mouse model.

(A) and **(B)** Violin plots of three ILC groups changing over time post infection in the lung and gut.

Hu et al. Supplementary Figure 8

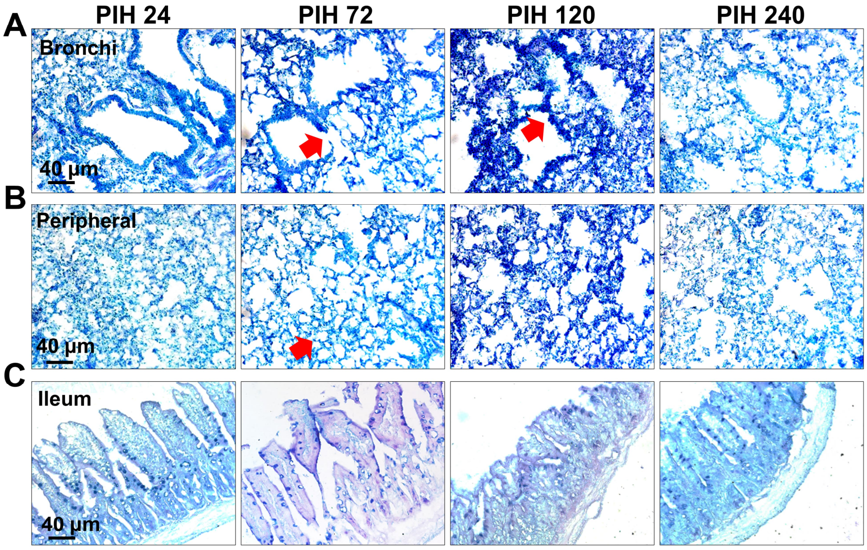

Supplementary Figure 8. AB-PAS staining of the lung and ileum of the COVID-19–like mouse model.

Goblet cells were stained in purple (arrow). **(A)** Dense goblet cells lined the bronchus. **(B)** Acidic mucins were disseminated in the peripheral alveolus. **(C)** Increase of goblet cells containing acidic mucins were seen in the ileum, especially at PIH 72.

Hu et al. Supplementary Figure 9

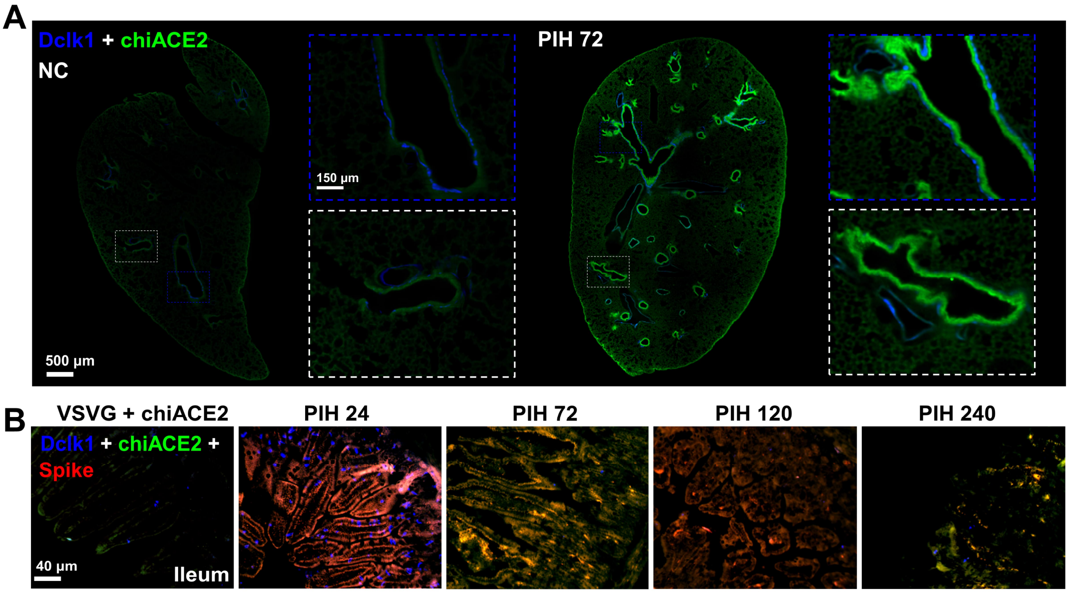

Supplementary Figure 9. tuft cells increased in response to the PSV-S in the lining of the lung-gut mucosa during the early phase of infection in the COVID-19–like mouse model.

(A) The blue square area of the bronchus showed an increase in tuft cells at PIH 72, while the white square area did not, indicating spatial heterogeneity of tuft cells. **(B)** Tuft cells increased extensively during PIH 24–72 and were co-localized with chiACE2 and PSV-S in the mucosa of the ileum.

Hu et al. Supplementary Figure 10

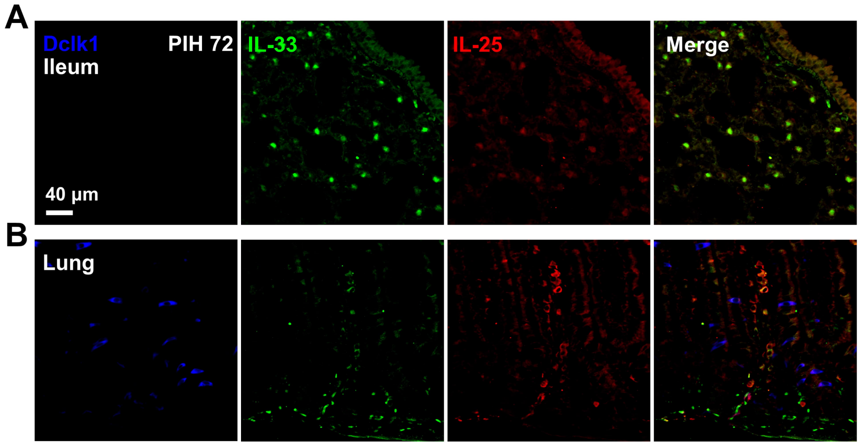

Supplementary Figure 10. Tuft cells and the differential expression of IL-25 and IL-33 in the mucosa of the lung and ileum of the COVID-19–like mouse model. Changes in IL-25 and IL-33 in the ileum post-infection. **(A)** and **(B)** IL-25 was widespread in the villi, with some foci colocalizing with tuft cells and some not, while IL-33 was mainly dispersed in the submucosa of the peripheral lung and ileum, with almost no colocalization with tuft cells.

Hu et al. Supplementary Figure 11

A

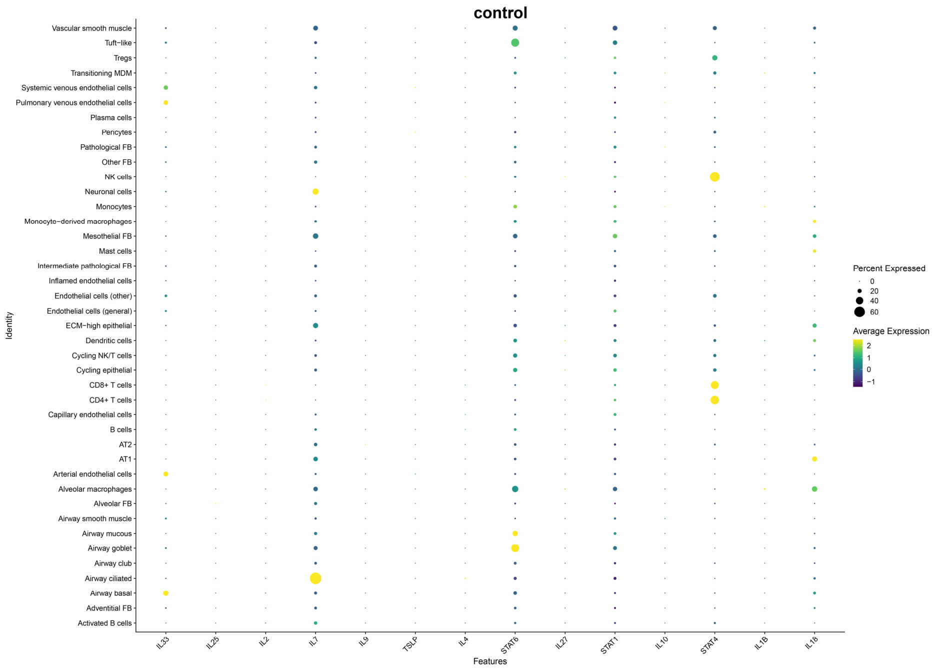

B

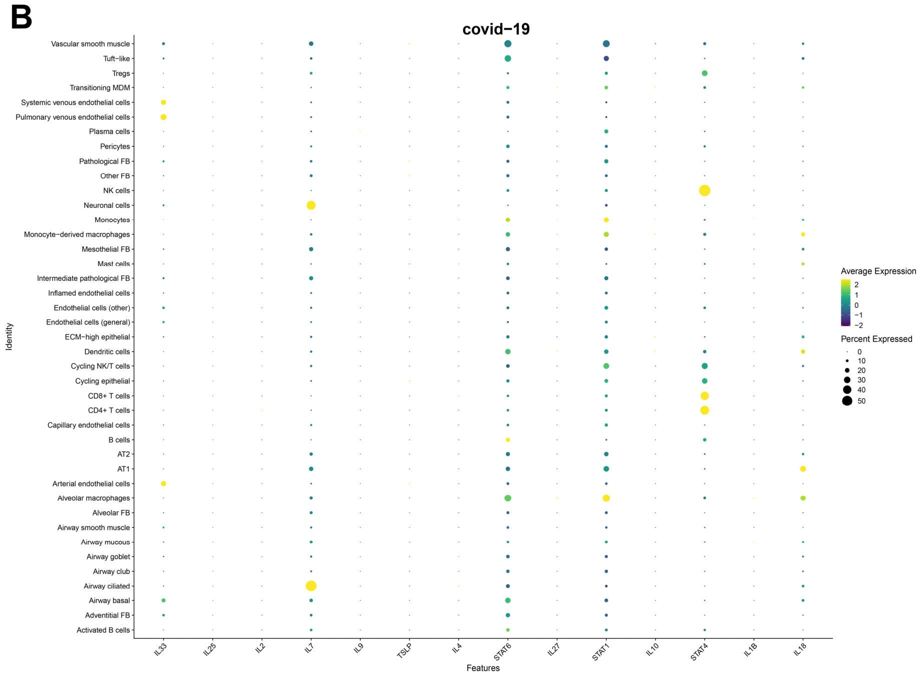

Supplementary Figure 11. Reanalysis of scRNA-seq data from COVID-19 patients. (A) and **(B)** The expression levels of tuft cell-related markers in major pulmonary cell types in controls and COVID-19 patients.

Hu et al. Supplementary Figure 12

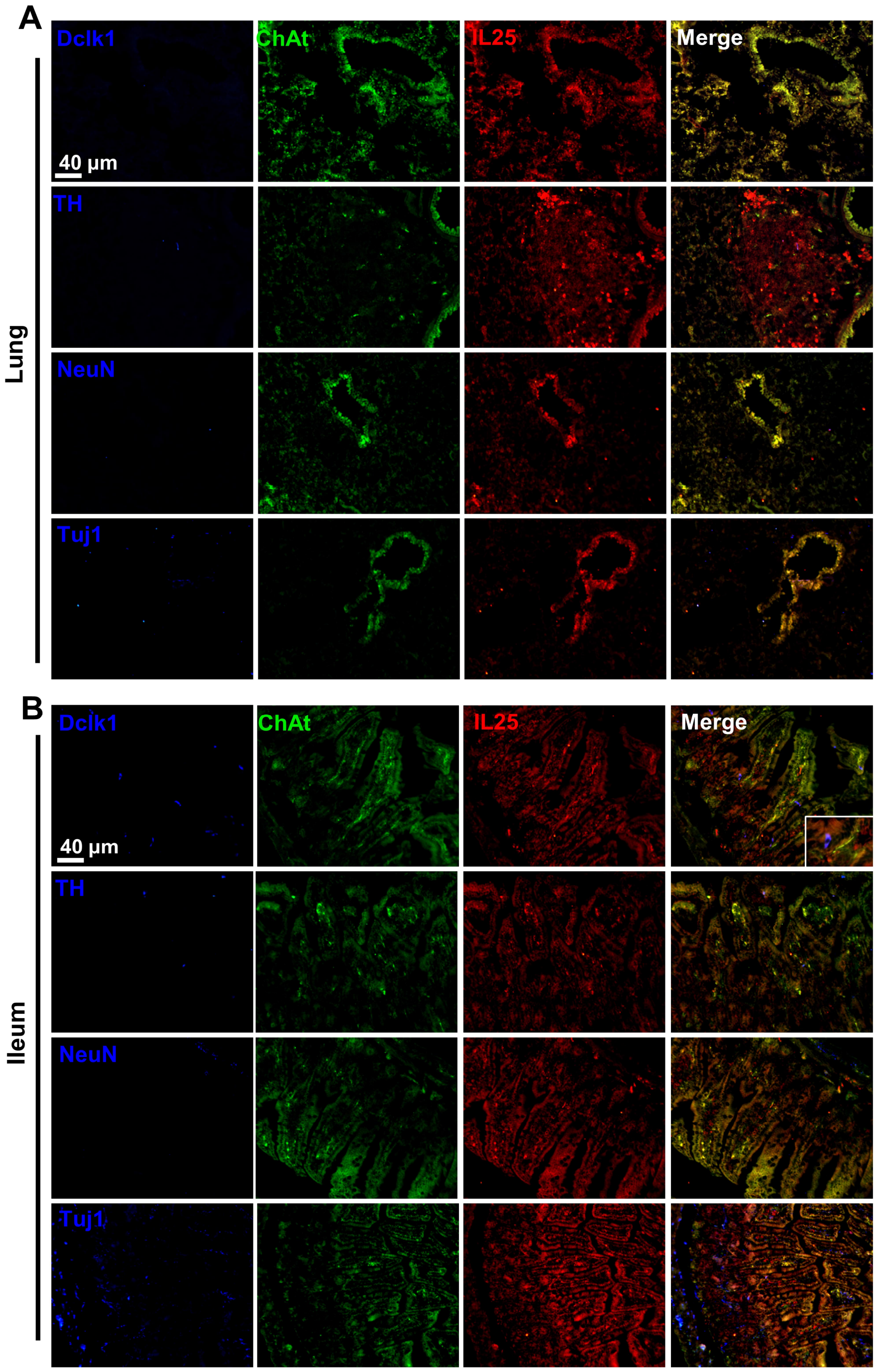

Supplementary Figure 12. The autonomic nervous system in relationship to tuft cells in the COVID-19–like mouse model.

(A) and **(B)** Autonomic neural markers, including sympathetic nerves (tyrosine hydroxylase-positive), parasympathetic nerves ($ChAt^{+}$), and neurons ($Tuji^{+}$) in the lung and ileum. The enlarged image shows an $IL25^{+}/ChAT^{+}$ nerve (red and green merged) approaching a tuft cell (blue).

Hu et al. Supplementary Figure 13

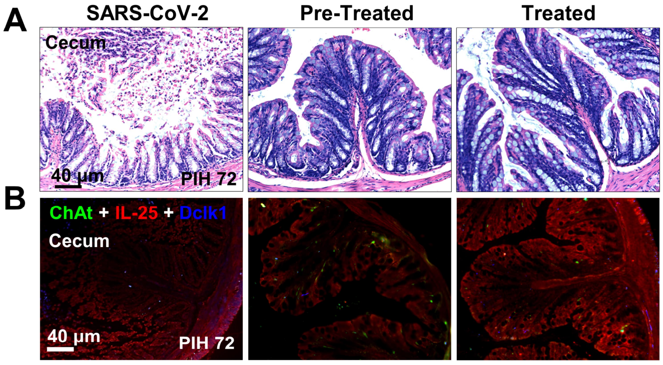

Supplementary Figure 13. Cholinergic modulation and tuft cells in the caecum of SARS-CoV-2-infected mice treated with unpublished neutralized antibodies.

The figure is divided into three columns, representing SARS-CoV-2-infected, pre-treated, and treated mice with unpublished neutralized antibodies, respectively. **(A)** HE staining of the caecum, revealing a more pronounced proliferation of goblet cells in pre-treated and treated mice compared to SARS-CoV-2-infected mice. **(B)** A greater degree of cholinergic modulation, a more intense type 2 immune response, and increased proliferation of tuft cells in pre-treated and treated mice when compared to SARS-CoV-2-infected mice.

Hu et al. Supplementary Figure 14

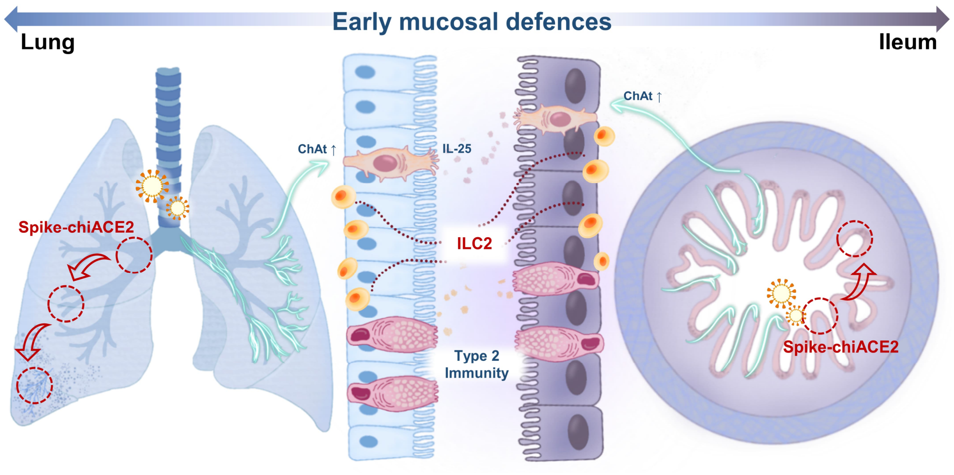

Supplementary Figure 14. Summary for Spatiotemporal observations of host-pathogen interactions in mucosa during SARS-CoV-2 infection

The spike protein of SARS-CoV-2 has the ability to bind to ACE2, facilitating the transduction of PSV-S through the mucosal barrier and its widespread diffusion into the alveoli or crypts within a span of 72 hours. During this process, ILC2s and type 2 immunity play a crucial role as the primary line of defence, supported by concurrent increases in tuft cells and cholinergic innervations, which are closely associated with this immune response.
